# Supplementary material for: Chemical tools to define and manipulate interferon-inducible Ubl protease USP18
Source: Nat Commun. 2025 Jan 22;16:957. doi: 10.1038/s41467-025-56336-5 (PMC11754618; doi:10.1038/s41467-025-56336-5)

Supplementary Information

Chemical tools to define and manipulate interferon-inducible Ubl protease USP18

Griffin J. Davis,[1,#] Anthony O. Omole,[1,#] Yejin Jung,[1,#] Wioletta Rut,[2] Ronald Holewinski,[3] Kiall F. Suazo,[3] Hong-Rae Kim,[1,4] Mo Yang,[1] Thorkell Andresson,[3] Marcin Drag,[2] Euna Yoo[1,*]

[1]Chemical Biology Laboratory, Center for Cancer Research, National Cancer Institute, National Institutes of Health, Frederick, Maryland 21702, United States
[2]Department of Chemical Biology and Bioimaging, Wroclaw University of Science and Technology, 50-370 Wroclaw, Poland
[3]Laboratory of Proteomics and Analytical Technologies, Frederick National Laboratory for Cancer Research, Leidos Biomedical Research, Frederick, Maryland 21702, United States
[4]Present address: Department of Biomedical Sciences, College of Medicine, Korea University, Seoul 02708, Korea

[#]These authors contributed equally to this work.
*Correspondence: euna.yoo@nih.gov

Supplementary figures

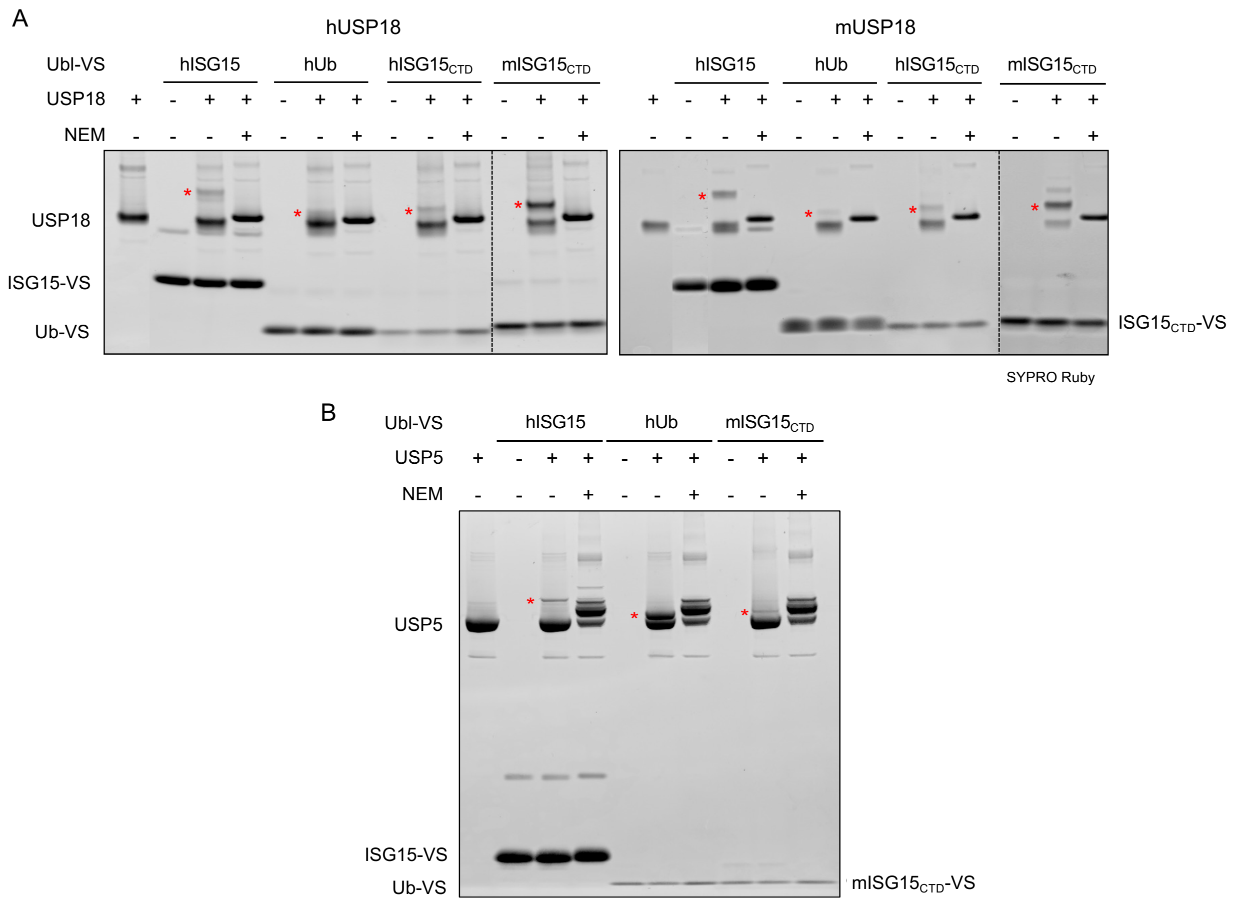

Supplementary Figure 1. Reactivity Ub-VS and ISG15-VS towards USP18 and USP5. (A) Recombinant human and mouse USP18 (2 μM) was incubated with hISG15-VS (5 μM), hUb-VS (20 μM), h/mISG15$_{CTD}$-VS (10 μM) for 3 h at RT. The result is a representative of two experiments (n = 2 independent replicates). (B) Recombinant human USP5 (1.5 μM) was incubated with each Ubl-VS (5 μM) for 3 h at RT. NEM pretreatment (10 μM for 10 min at RT) was performed where indicated. Protein samples were analyzed by SDS-PAGE and SYPRO Ruby staining. The result is a representative of two experiments (n = 2 independent replicates).

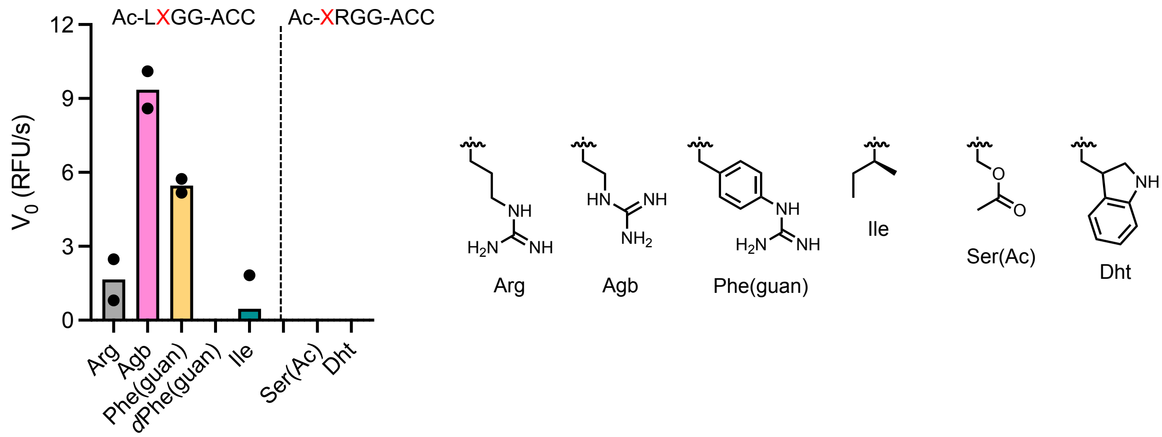

Supplementary Figure 2. Rate of hydrolysis of selected tetrapeptide fluorogenic substrates. mUSP18 (10 μM) was incubated with 100 μM of substrates for 1 h at RT and the initial release of fluorescent ACC (V_0, RFU/s) by enzyme was measured at Ex: 360 nm / Em: 460 nm. Data represent mean values (n = 2 independent replicates). Source data are provided as a Source Data file.

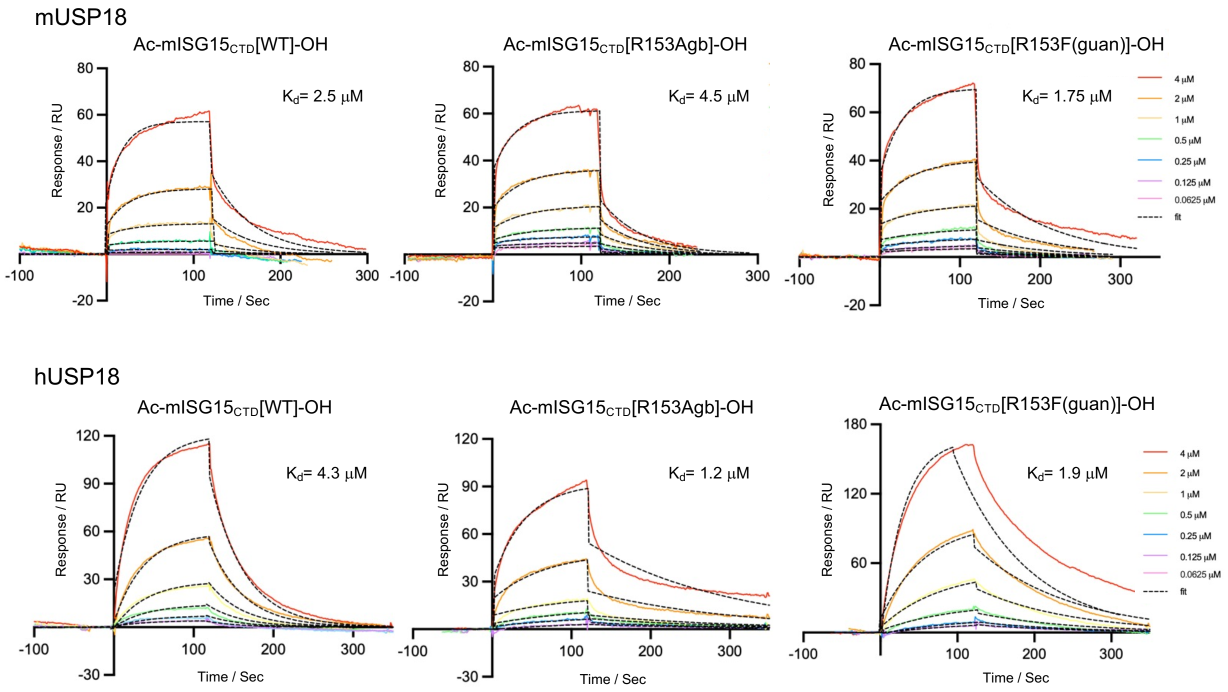

Supplementary Figure 3. SPR analysis of the USP18-ISG15 interaction. Mouse or human USP18 was immobilized on Ni-NTA chips and association and dissociation of $mISG15_{CTD}$ variants were monitored.

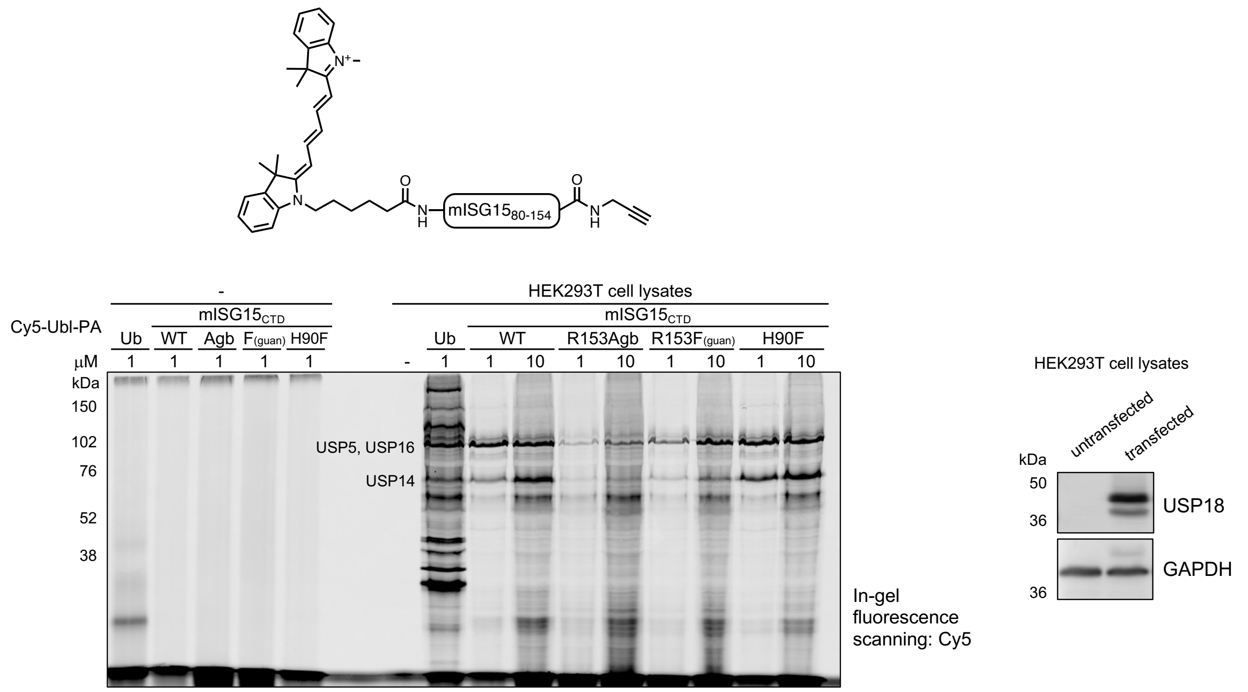

Supplementary Figure 4. Selectivity of mISG15$_{CTD}$-based probes. HEK293T cell lysates were incubated with Cy5-mISG15$_{CTD}$-PA probes for 3 h at RT. Protein samples were analyzed by SDS-PAGE and in-gel fluorescence scanning for Cy5 signal. Immunoblotting data indicated the absence of USP18 detected in HEK293T cell lysates in comparison to HEK293T cells transfected with USP18-FLAG. The result is a representative of three experiments (n = 3 independent replicates). Source data are provided as a Source Data file.

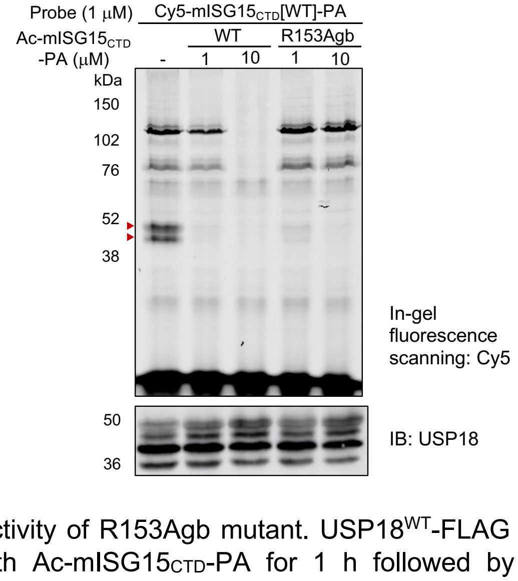

Supplementary Figure 5. Selectivity of R153Agb mutant. USP18^{WT}-FLAG overexpressing HEK293T cell lysates were preincubated with Ac-mISG15$_{CTD}$-PA for 1 h followed by labeling with 1 μM of Cy5-mISG15$_{CTD}$[WT]-PA for 2 h at RT to measure the selective competition for USP18 by the Agb probe. Protein samples were analyzed by SDS-PAGE and in-gel fluorescence scanning for Cy5 signal. Red marks correspond to the expected molecular weight of USP18–probe conjugate. The result is a representative of two experiments (n = 2 independent replicates). Source data are provided as a Source Data file.

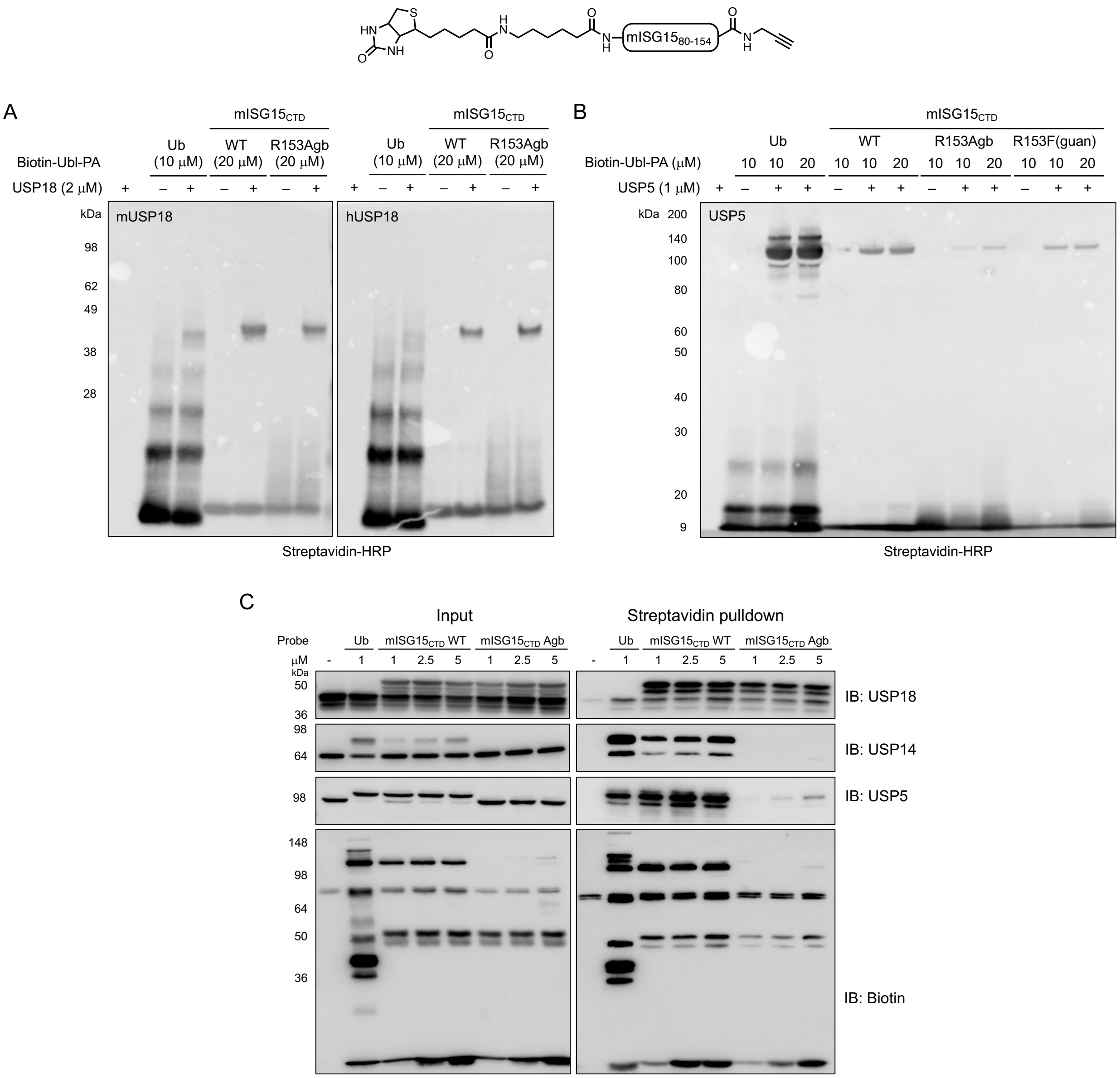

Supplementary Figure 6. Reactivity and selectivity of Biotin-mISG15$_{CTD}$-based probes. (A-B) Recombinant human and mouse USP18 (2 μM) or USP5 (1 μM) was incubated with Biotin-mISG15$_{CTD}$-PA probes at indicated concentrations for 3 h at RT. Protein samples were analyzed by SDS-PAGE and far-western blotting using streptavidin-HRP. The result is a representative of two experiments (n = 2 independent replicates). (C) USP18-FLAG overexpressing HEK293T cell lysates were incubated with Biotin-mISG15$_{CTD}$-PA probes at indicated concentrations for 3 h at RT. Biotinylated proteins were enriched by streptavidin pulldown and analyzed by SDS-PAGE and immunoblotting. The result is a representative of two experiments (n = 2 independent replicates). Source data are provided as a Source Data file.

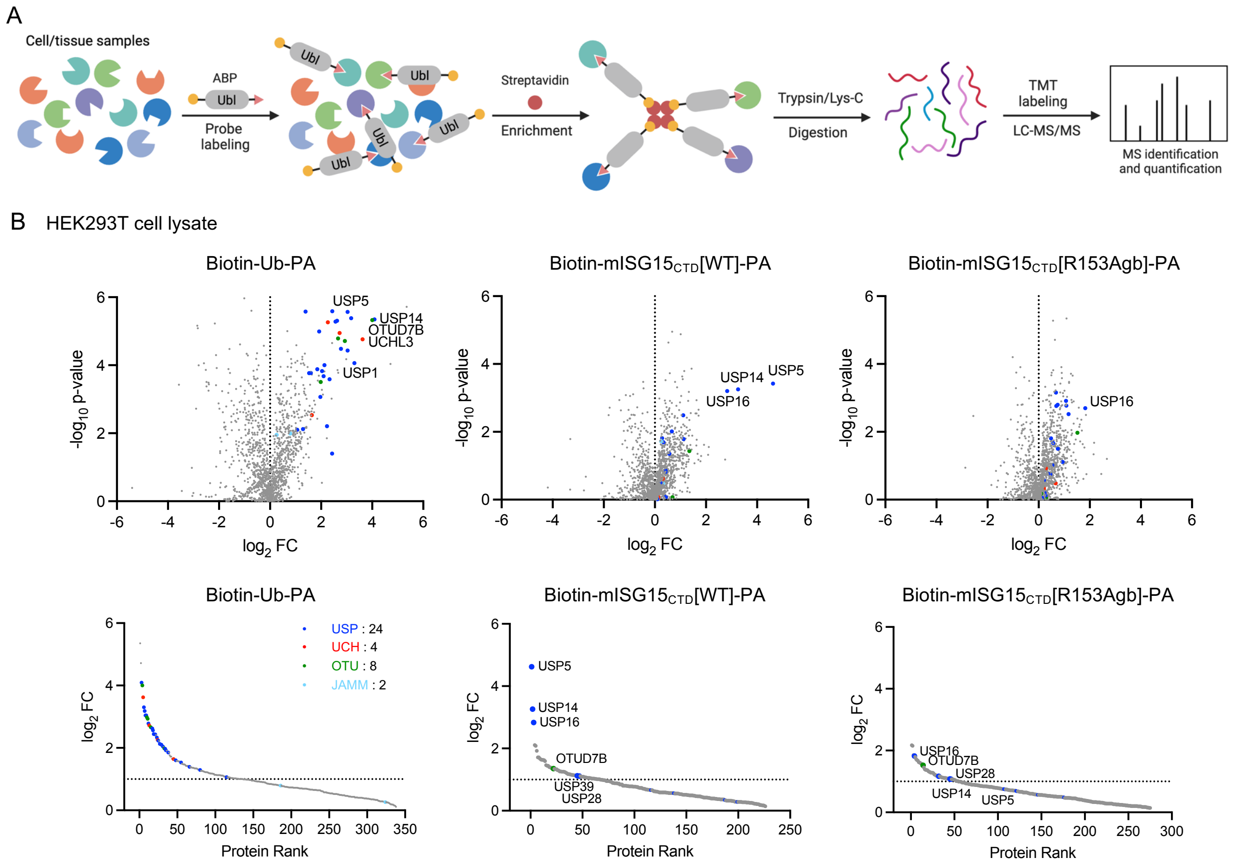

Supplementary Figure 7. Activity-based protein profiling by Biotin-mISG15CTD-PA probes. (A) Schematic workflow for identification of Ubl proteases in cell lysates created in BioRender. Yoo, E. (2025) https://BioRender.com/p15i571. (B) Volcano (top) and protein rank (bottom) plots of quantitative proteomic analysis of streptavidin beads pulldowns after labeling of HEK 293T cell lysates by Biotin-Ub-PA, Biotin-mISG15CTD[WT]-PA or Biotin-mISG15CTD[R153Agb]-PA probes (1 μM, 2 h, RT) showing significantly enriched proteins (log_2 ratio > 1, p-value ≤ 0.05). DUBs are colored based on subfamilies (blue: USP, red: UCH, green: OUT, and cyan: JAMM). Data represent mean values (n = 3 independent replicates). p-values were calculated by two-tailed *t* test. Source data are provided with this paper (Supplementary Data 1).

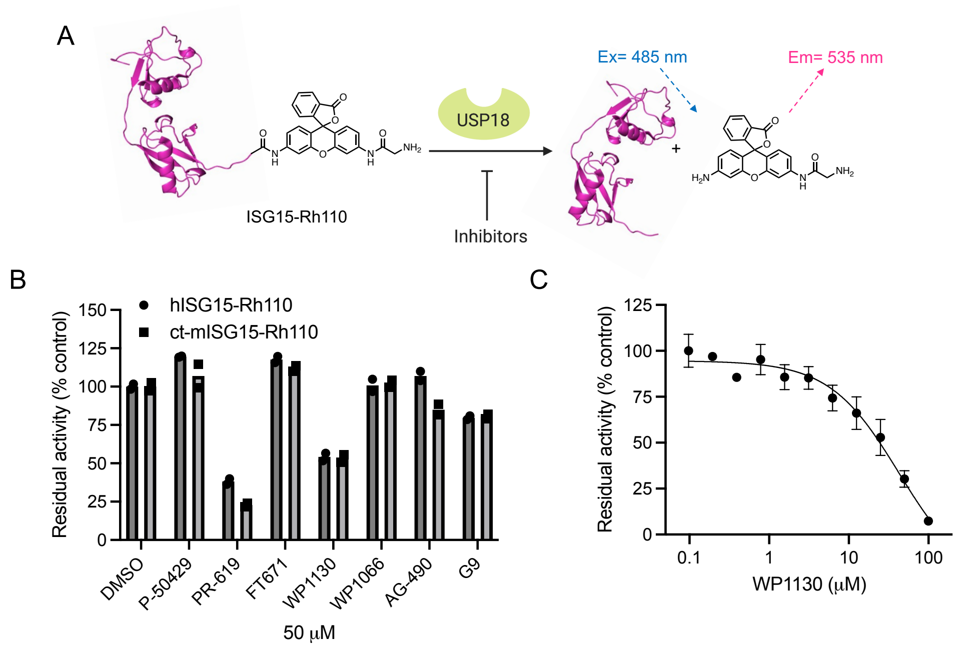

Supplementary Figure 8. USP18 biochemical assay. (A) Schematic of ISG15-Rho screening assay. (B) Screening of known inhibitors of DUBs. Recombinant mouse USP18 (2.5 nM) was incubated with 50 μM of each compound. The residual USP18 activity was measured for the cleavage of a rhodamine-based fluorogenic substrate read at Ex. 485 / Em. 535 nM and normalized to DMSO control. Data represent mean values (n = 2 independent replicates). Source data are provided as a Source Data file. (C) IC_{50} curve of WP1130 for USP18 inhibition. Data represent mean values ± SD (n = 2 independent replicates). Source data are provided as a Source Data file.

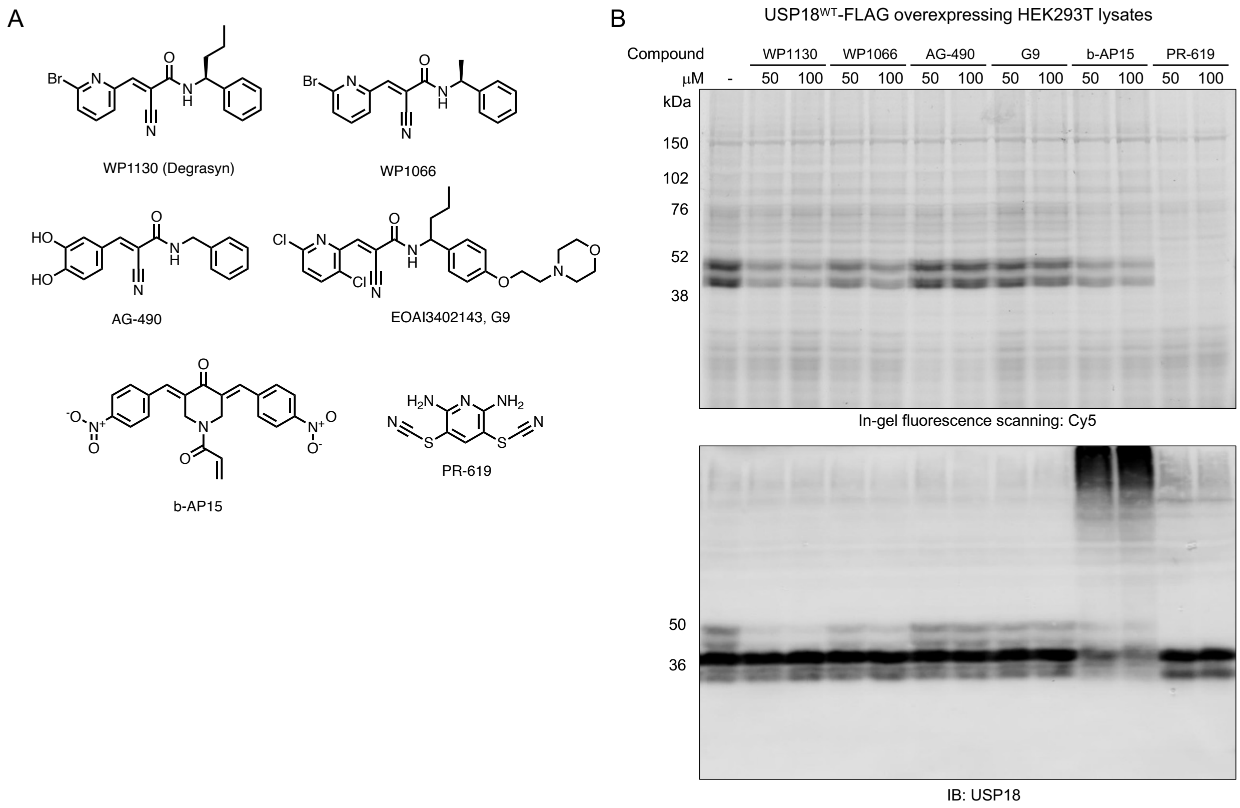

Supplementary Figure 9. Gel-based ABPP with DUB inhibitors. (A) Chemical structure of DUB inhibitors tested. (B) USP18^{WT}-FLAG overexpressing HEK293T cell lysates were preincubated with each compound at indicated concentration for 1 h followed by labeling with 1 μM of Cy5-Ubl-PA for 2 h at RT. Protein samples were analyzed by SDS-PAGE, in-gel fluorescence scanning for Cy5 signal, and immunoblotting for USP18. The result is a representative of two experiments (n = 2 independent replicates). Source data are provided as a Source Data file.

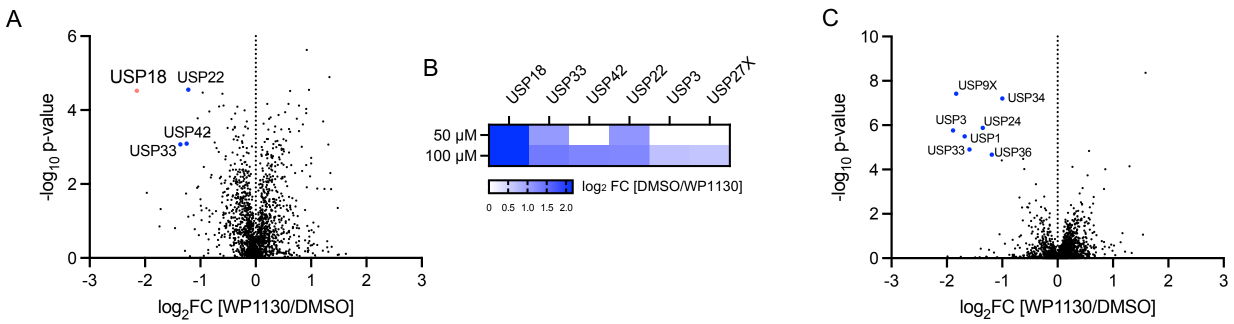

Supplementary Figure 10. Competitive ABPP for DUB inhibitor screening. (A-B) USP18^{WT}-FLAG overexpressing HEK293T cell lysates were preincubated with 100 μM of WP1130 for 2 h followed by labeling with a cocktail of probes (1 μM of Biotin-Ub-PA + 5 μM of Biotin-mISG15$_{CTD}$[R153Agb]-PA) for 3 h at RT. Protein samples were enriched by streptavidin, on-bead digested, and analyzed by LC-MS/MS after TMT labeling. (A) A volcano plot of quantitative proteomic analysis comparing samples treated with 50 μM of WP1130 to DMSO is shown with DUBs inhibited by the compound marked. USP18 is marked and other DUBs are colored based on subfamilies. Data represent mean values (n = 3 independent replicates). p-values were calculated by two-tailed *t* test. Source data are provided with this paper (Supplementary Data 2). (B) Heat map analysis of log$_2$ fold change of MS intensity for competitively enriched proteins comparing samples pretreated with 50 μM or 100 μM of WP1130 to DMSO samples. Data represent mean values (n = 3 independent replicates). Source data are provided as a Source Data file. (C) HEK293T cell lysates were preincubated with 50 μM of WP1130 for 2 h followed by labeling with 1 μM of Biotin-Ub-PA for 3 h at RT. Protein samples were enriched by streptavidin, on-bead digested, and analyzed by LC-MS/MS after TMT labeling. A volcano plot of quantitative proteomic analysis comparing samples treated with 50 μM of WP1130 to DMSO is shown with DUBs inhibited by the compound marked. Data represent mean values (n = 3 independent replicates). p-values were calculated by two-tailed *t* test. Source data are provided with this paper (Supplementary Data 3).

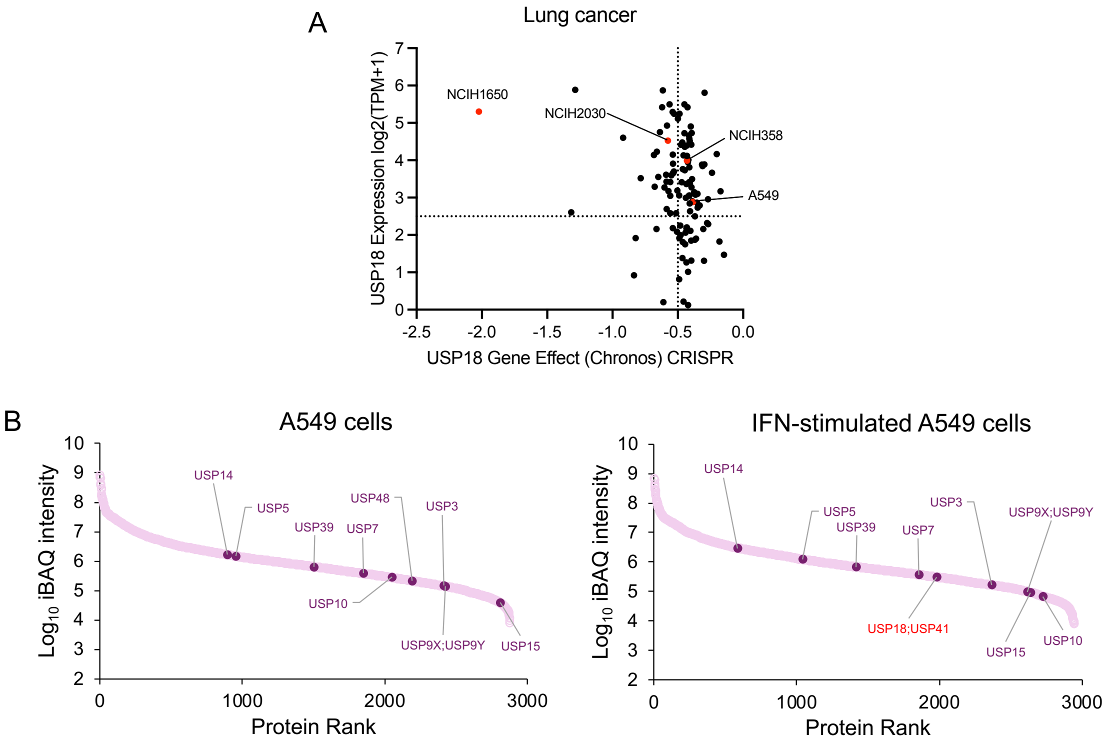

Supplementary Figure 11. (A) Analysis of lung cancer cell lines for USP18 expression and gene effect (CRISPR) from the Broad DepMap database. Red dots are cell lines used in this study. Source data are provided as a Source Data file. (B) Relative abundance of DUBs determined by intensity-based absolute quantification (iBAQ) proteomics in A549 cells. Data represent mean values (n = 3 independent replicates).

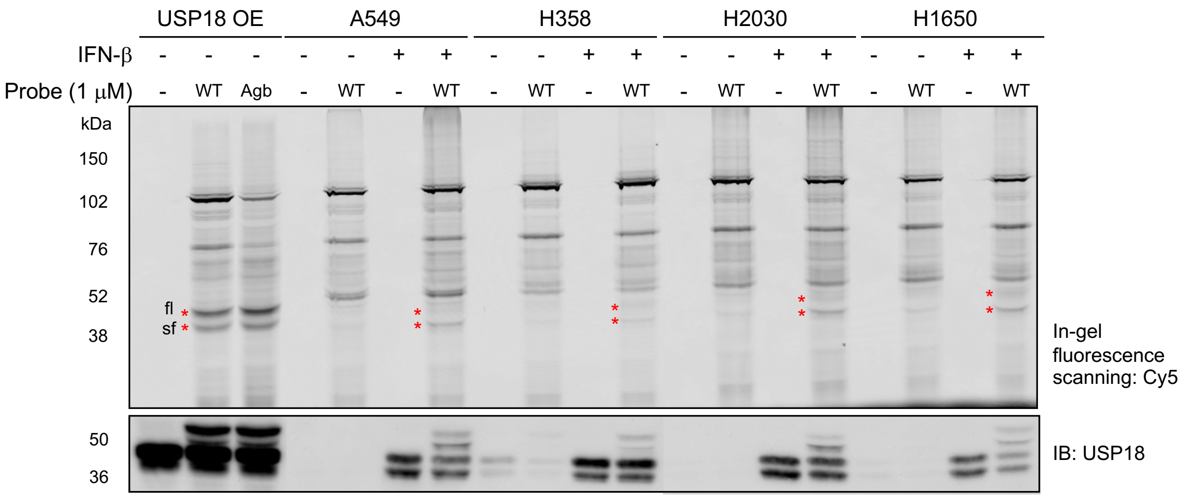

Supplementary Figure 12. Gel-based ABPP in lung cancer cell lines. Each cell lysates were incubated with 1 µM of Cy5-mISG15$_{CTD}$[WT]-PA probes for 3 h at RT. Protein samples were analyzed by SDS-PAGE and in-gel fluorescence scanning for Cy5 signal. Red marks indicate the appearance of USP18–probe conjugates. Expression of USP18 was confirmed by western blotting. The result is a representative of three experiments (n = 3 independent replicates). Source data are provided as a Source Data file.

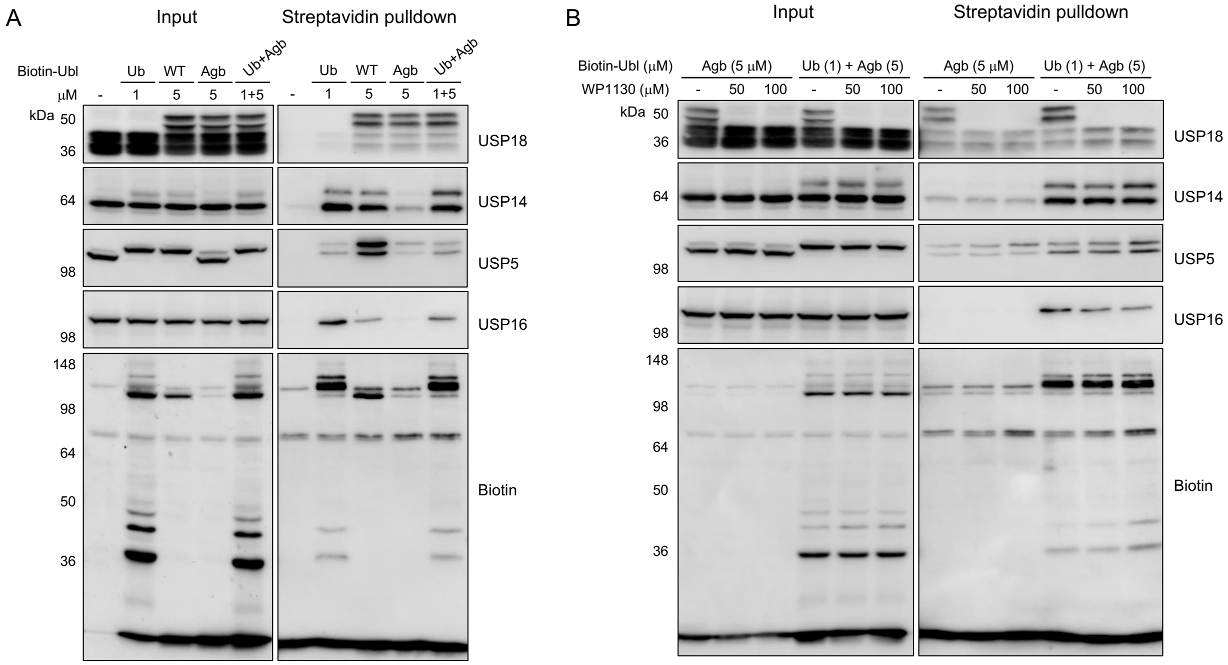

Supplementary Figure 13. Immunoblotting analysis of DUB ABPP in A549 cells. (A) Lysates of A549 cells stimulated with IFN-β (50 ng/mL, 48 h) were incubated with each Biotin-Ubl-PA probe at indicated concentrations for 3 h at RT. Biotinylated proteins were enriched by streptavidin pulldown and analyzed by SDS-PAGE and immunoblotting. The result is a representative of two experiments (n = 2 independent replicates). Source data are provided as a Source Data file. (B) Lysates of A549 cells stimulated with IFN-β (50 ng/mL, 48 h) were preincubated with WP1130 at indicated concentrations for 2 h followed by labeling with 5 μM of Biotin-mISG15$_{CTD}$[R153Agb]-PA or a cocktail of probes (1 μM of Biotin-Ub-PA + 5 μM of Biotin-mISG15$_{CTD}$[R153Agb]-PA) for 3 h at RT. Competitively biotinylated proteins were enriched by streptavidin pulldown and analyzed by SDS-PAGE and immunoblotting. The result is a representative of two experiments (n = 2 independent replicates). Source data are provided as a Source Data file.

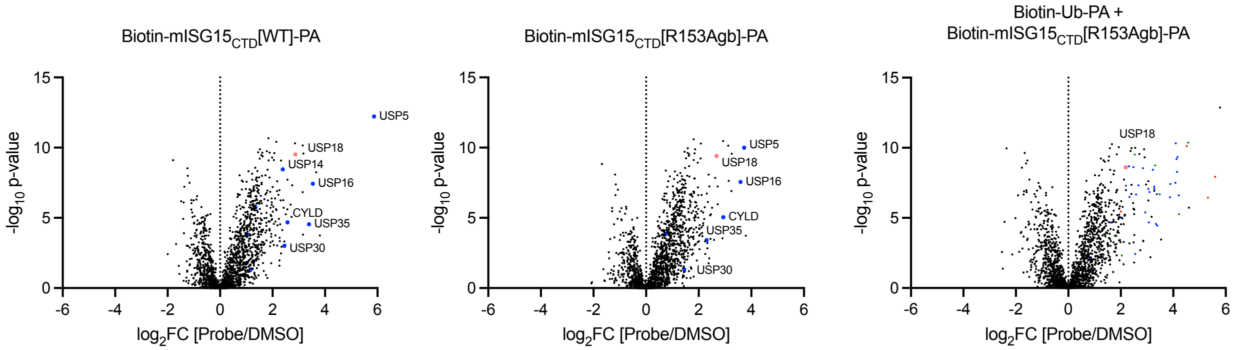

Supplementary Figure 14. Volcano plots of quantitative proteomic analysis comparing samples treated with each Biotin-Ubl-PA probe to DMSO. Lysates of A549 cells stimulated with IFN-β (50 ng/mL, 48 h) were incubated with either 5 μM of Biotin-mISG15$_{CTD}$[WT]-PA (left), Biotin-mISG15$_{CTD}$[R153Agb]-PA (middle) or a cocktail of probes (1 μM of Biotin-Ub-PA + 5 μM of Biotin-mISG15$_{CTD}$[R153Agb]-PA) (right) for 3 h at RT. Protein samples were enriched by streptavidin, on-bead digested, and analyzed by LC-MS/MS after TMT labeling. Data represent mean values (n = 3 independent replicates). p-values were calculated by two-tailed *t* test. Source data are provided with this paper (Supplementary Data 4).

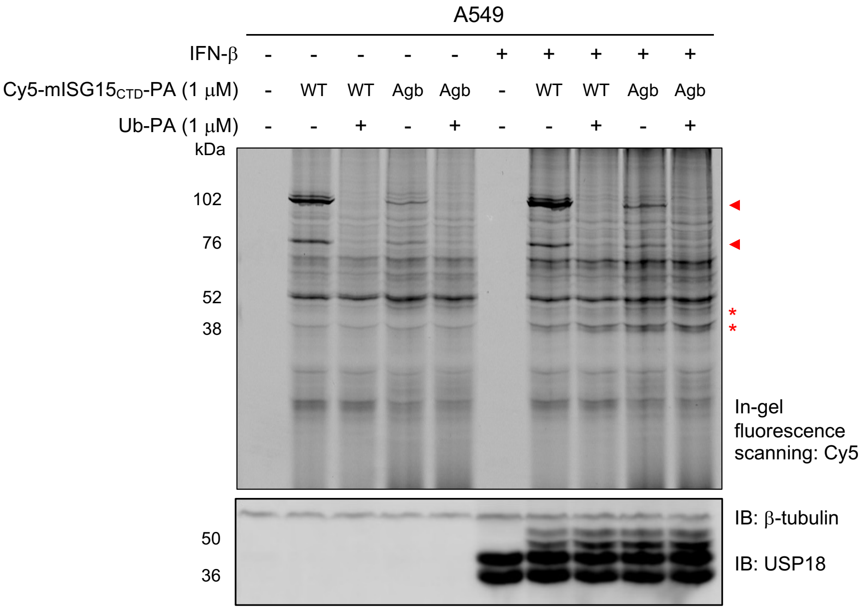

Supplementary Figure 15. Competitive ABPP. Lysates of A549 cells were preincubated with Ub-PA (1 μM) for 1 h followed by labeling with 1 μM of either Cy5-mISG15$_{CTD}$[WT]-PA or Cy5-mISG15$_{CTD}$[R153Agb]-PA for 3 h at RT to measure the selective competition for USP18 by the Agb probe. Protein samples were analyzed by SDS-PAGE and in-gel fluorescence scanning for Cy5 signal. Red arrows indicate DUBs that are significantly blocked by preincubation with Ub-PA and red asterisk marks correspond to the expected molecular weight of USP18–probe conjugate. The result is a representative of two experiments (n = 2 independent replicates). Source data are provided as a Source Data file.

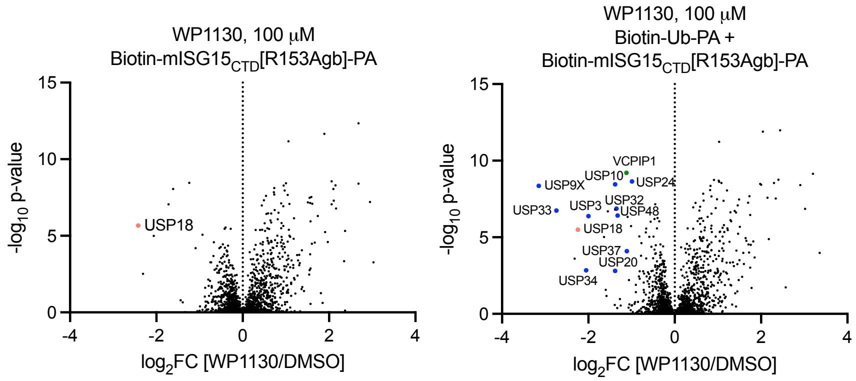

Supplementary Figure 16. Lysates of A549 cells stimulated with IFN-β were preincubated with 100 μM of WP1130 for 2 h followed by labeling with either 5 μM of Biotin-mISG15$_{CTD}$[R153Agb]-PA (left) or a cocktail of probes (1 μM of Biotin-Ub-PA + 5 μM of Biotin-mISG15$_{CTD}$[R153Agb]-PA) (right) for 3 h at RT. Volcano plots of quantitative proteomic analysis comparing samples treated with WP1130 to DMSO are shown with DUBs inhibited by the compound marked. Data represent mean values (n = 3 independent replicates). p-values were calculated by two-tailed *t* test. Source data are provided with this paper (Supplementary Data 5).

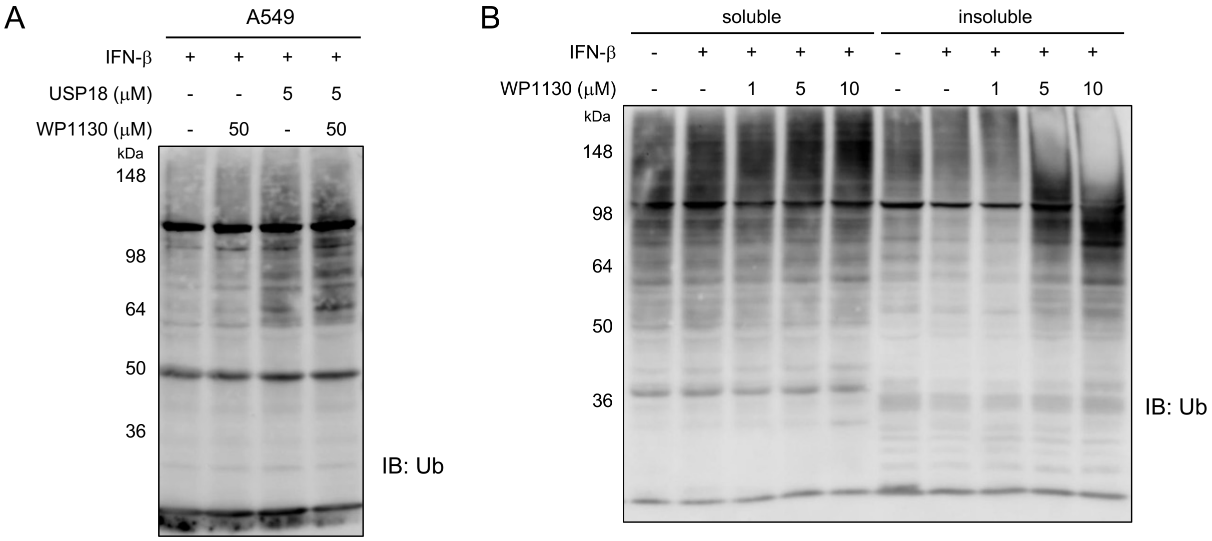

Supplementary Figure 17. (A) Lysates of A549 cells stimulated with IFN-β (48 h) were incubated with WP1130 (50 μM) for 2 h in the absence or presence of recombinant human USP18 (5 μM). Protein samples were analyzed by SDS-PAGE and immunoblotted for protein ubiquitylation. The result is a representative of three experiments (n = 3 independent replicates). Source data are provided as a Source Data file. (B) A549 cells were stimulated with IFN-β (48 h) and incubated with the indicated concentration of WP1130 for 2 h. Lysates were separated into soluble and insoluble fractions and probed for protein ubiquitylation. The result is a representative of three experiments (n = 3 independent replicates). Source data are provided as a Source Data file.

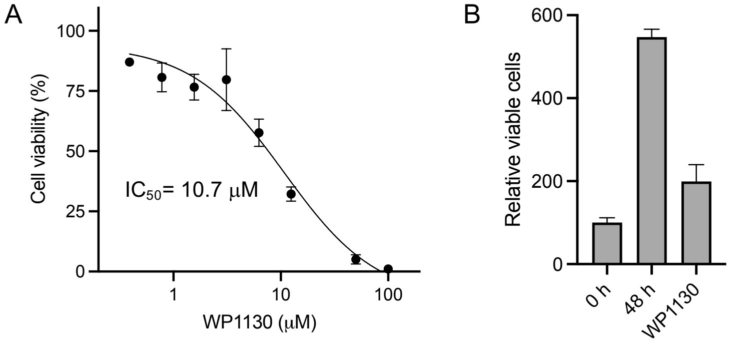

Supplementary Figure 18. Cell viability assay with WP1130. (A) A549 cells were incubated with increasing concentrations of WP1130 for 6 h and viable cells were determined and normalized to DMSO control. Data represent mean values ± SD (n = 3 independent replicates). Source data are provided as a Source Data file. (B) The antiproliferative effect of WP1130 (10 μM) was measured against A549 cells after 48 h treatment in the presence of IFN-β. Data represent mean values ± SD (n = 3 independent replicates).

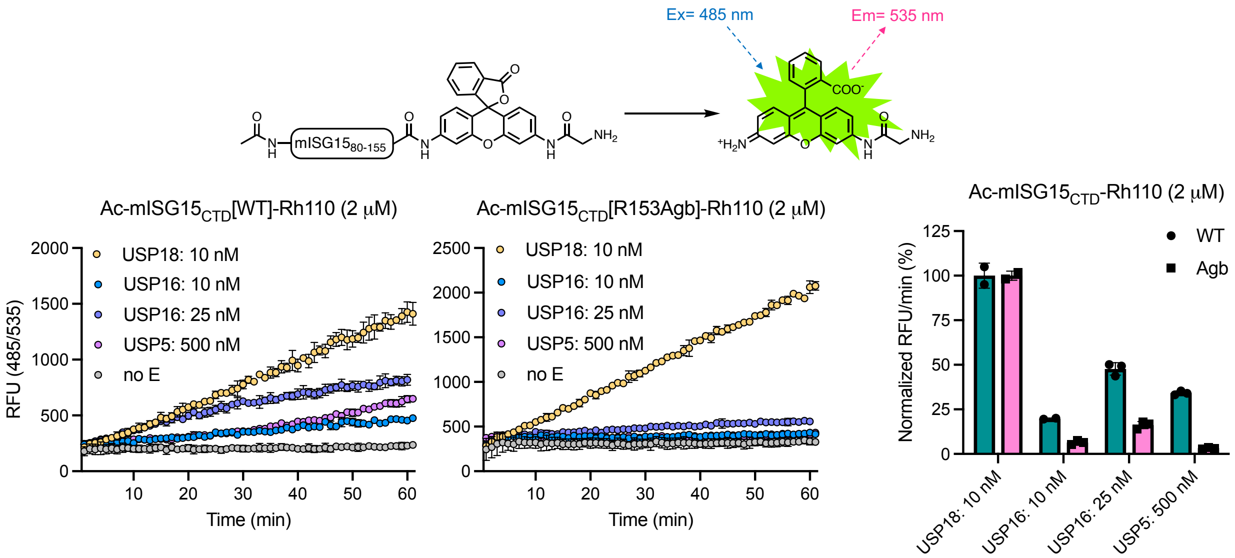

Supplementary Figure 19. ISG15CTD-Rho cleavage assay with USP16. USP16 activity was measured for the cleavage of a rhodamine-based fluorogenic substrate, either WT or R153Agb mutant of Ac-mISG15CTD-Rh110, read at Ex. 485 / Em. 535 nM and compared to USP18 or USP5. Data represent mean values ± SD (n = 3 independent replicates). Source data are provided as a Source Data file.

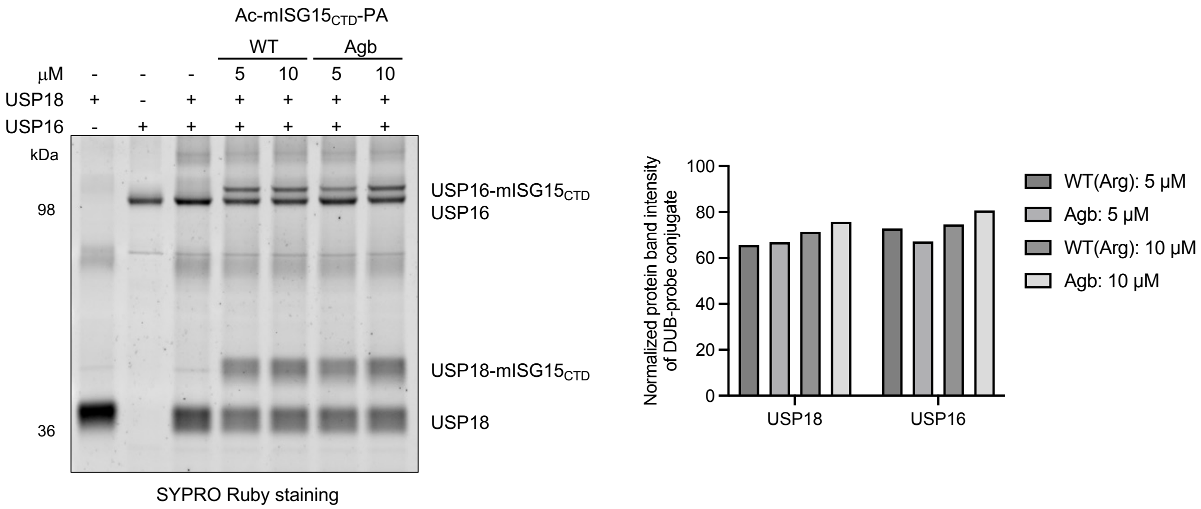

Supplementary Figure 20. Left, An equimolar mixture of recombinant human USP18 and USP16 was incubated with either Ac-mISG15CTD[WT]-PA or Ac-mISG15CTD[R153Agb]-PA at indicated concentrations for 3 h at RT. Protein samples were analyzed by SDS-PAGE and SYPRO Ruby staining. Right, Normalized mean density of labeled protein band corresponding to the USP–Ubl probe conjugate to mean density of unconjugated protein. The result is a representative of three experiments (n = 3 independent replicates). Source data are provided as a Source Data file.

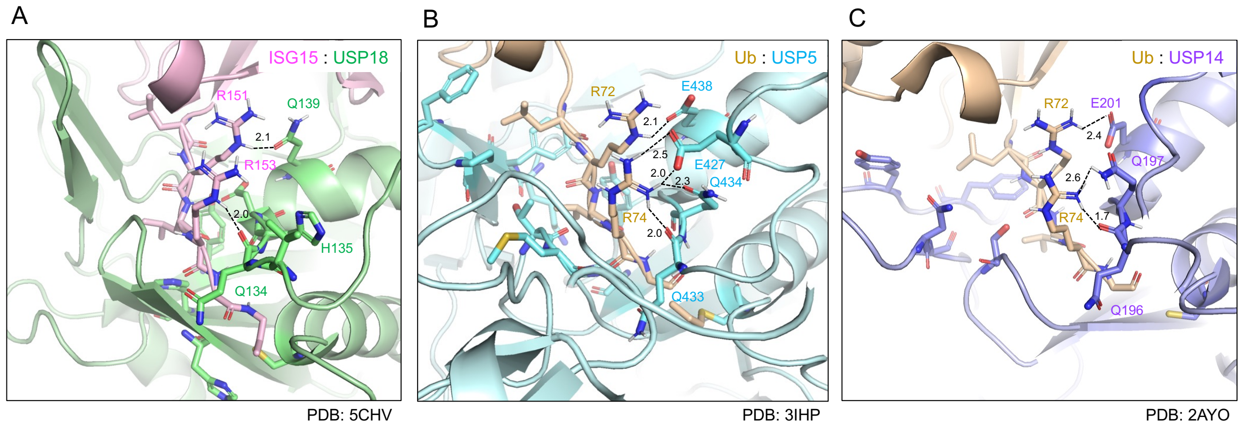

134QHDAAQ USP18
433QQDAQE USP5
196QQDANE USP14

Supplementary Figure 21. Structure analysis of ISG15 cross-reactive DUBs for the interaction with Ubl C-termini. Close-up view of the area where the C-terminal tail of Ubl (LRGG motif) is guided to the catalytic center of DUBs with (A) mUSP18 (green) in complex with mISG15-PA (pink), (B) USP5 (cyan) in complex with Ub-PA (wheat), (C) USP14 (purple) in complex with Ub-Ald (wheat). Various residues important for interaction are shown as sticks.

Analytical data

LC-MS

Ac-mISG15$_{CTD}$[WT]-OH

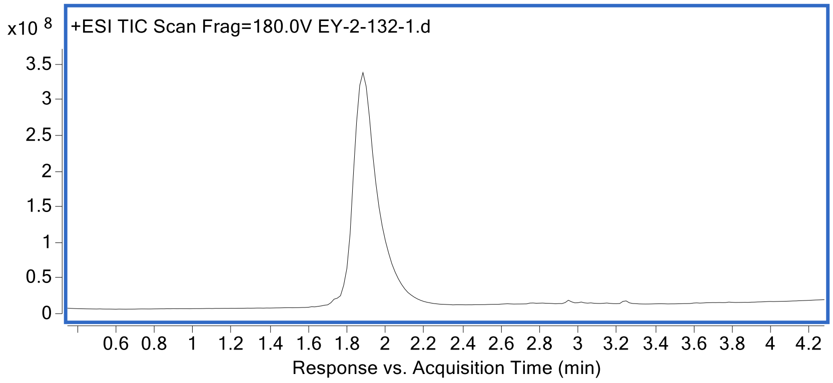

HRMS (ESI): m/z [M] calc. for $C_{399}H_{638}N_{114}O_{118}S$: 8952.2330, found 8952.71.

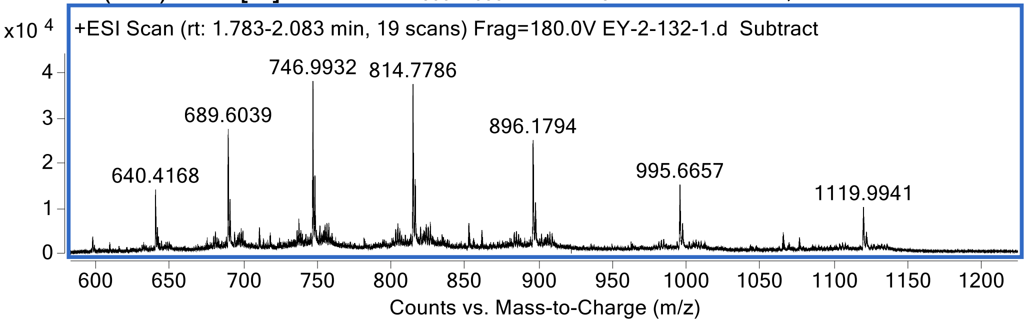

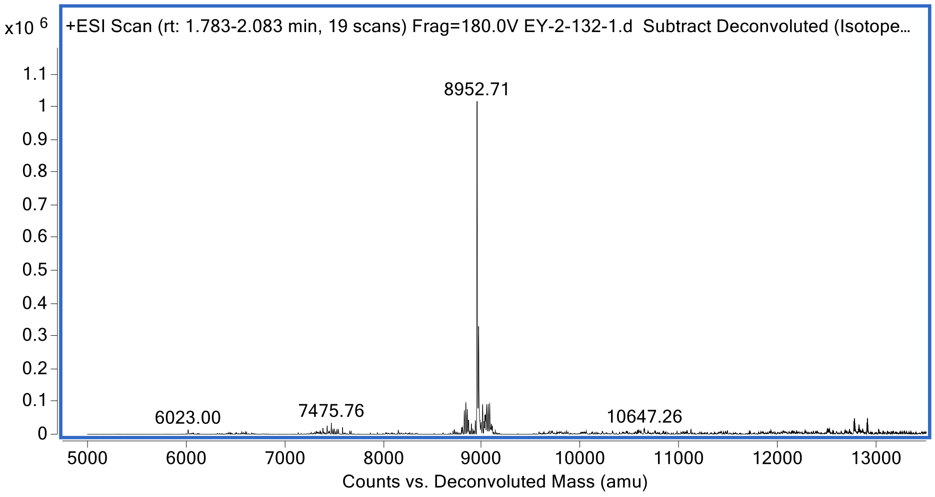

Ac-mISG15$_{CTD}$[R153Agb]-OH

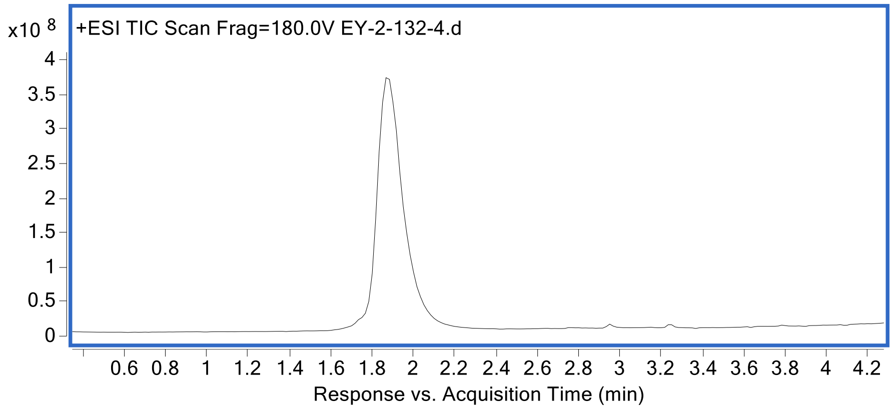

HRMS (ESI): m/z [M] calc. for $C_{398}H_{636}N_{114}O_{118}S$: 8938.2060, found 8938.54.

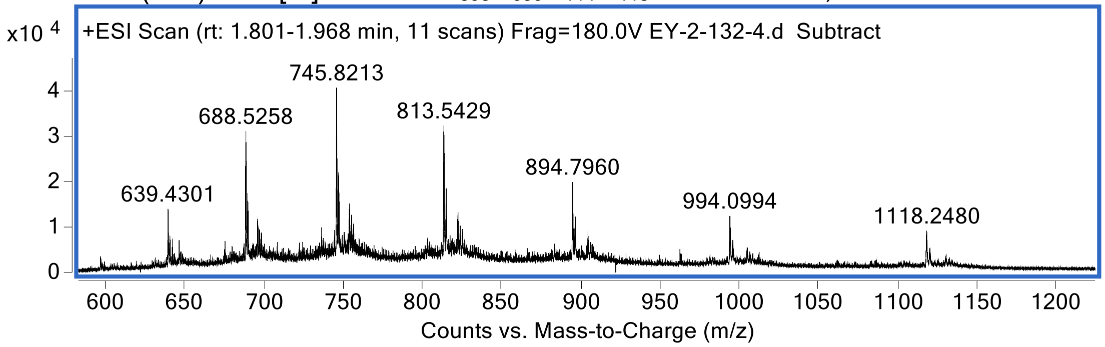

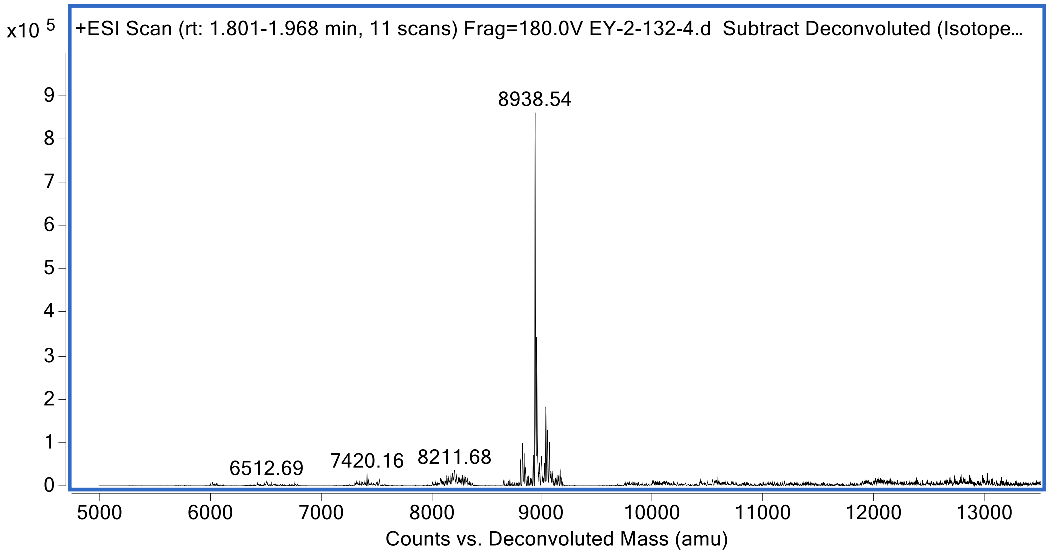

Ac-mISG15$_{CTD}$[R153F(guan)]-OH

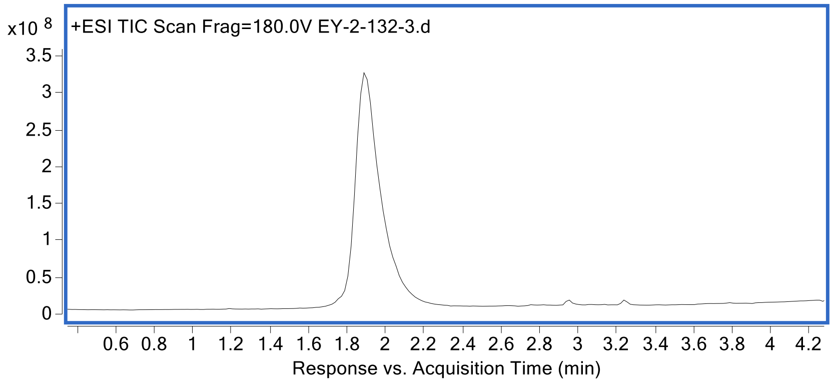

HRMS (ESI): m/z [M] calc. for $C_{403}H_{638}N_{114}O_{118}S$: 9000.2770, found 9000.68.

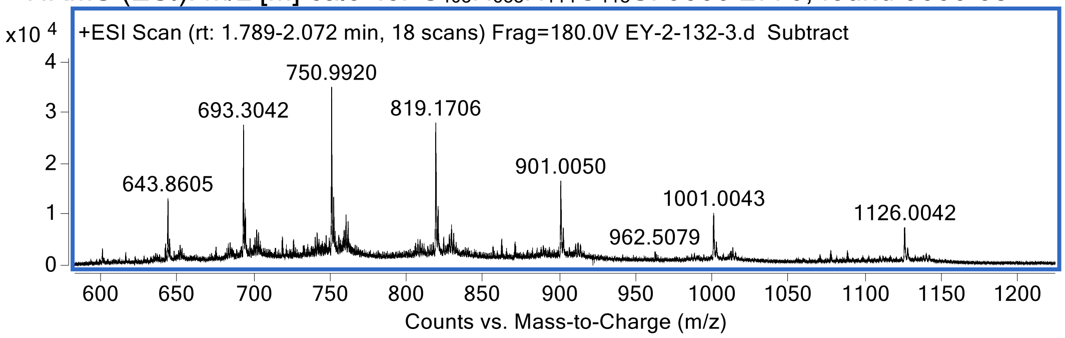

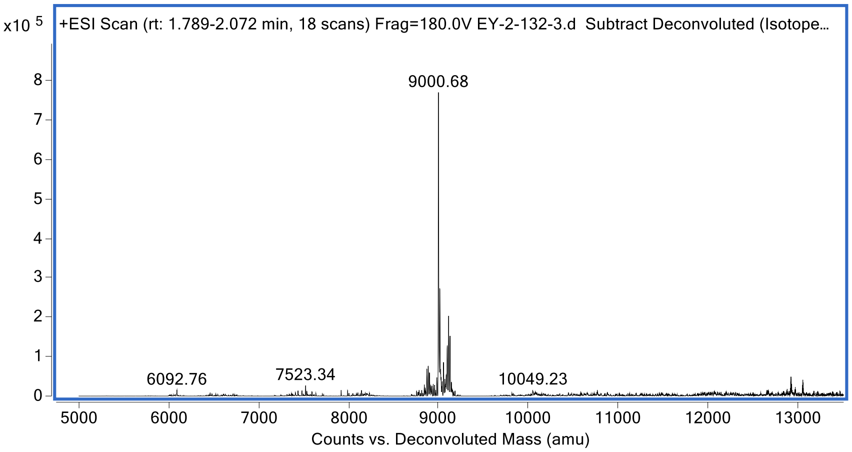

Ac-mISG15$_{CTD}$[WT]-PA

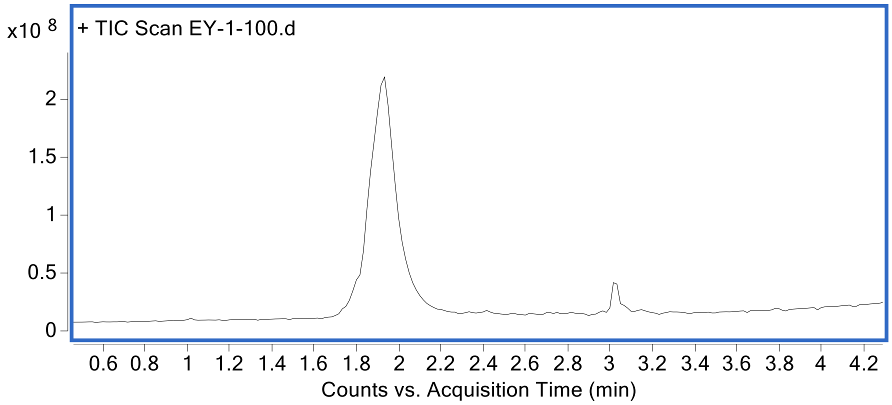

HRMS (ESI): m/z [M] calc. for $C_{400}H_{638}N_{114}O_{116}S$: 8932.25, found 8932.66.

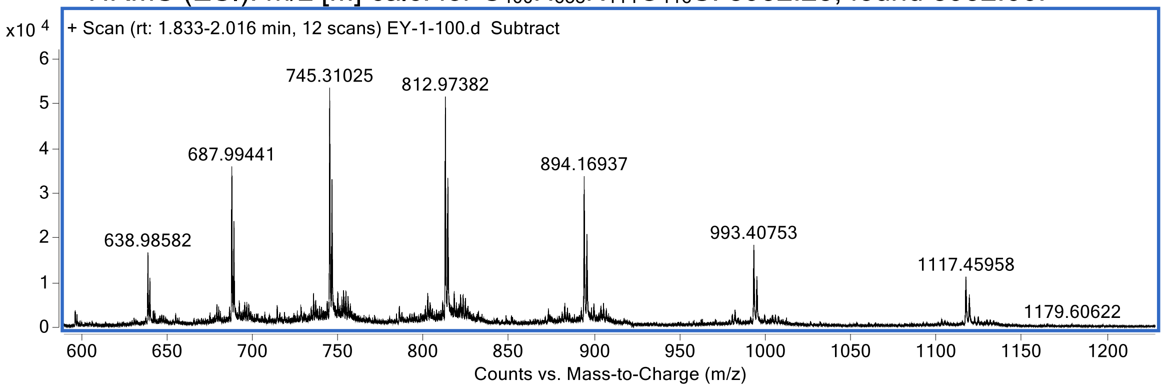

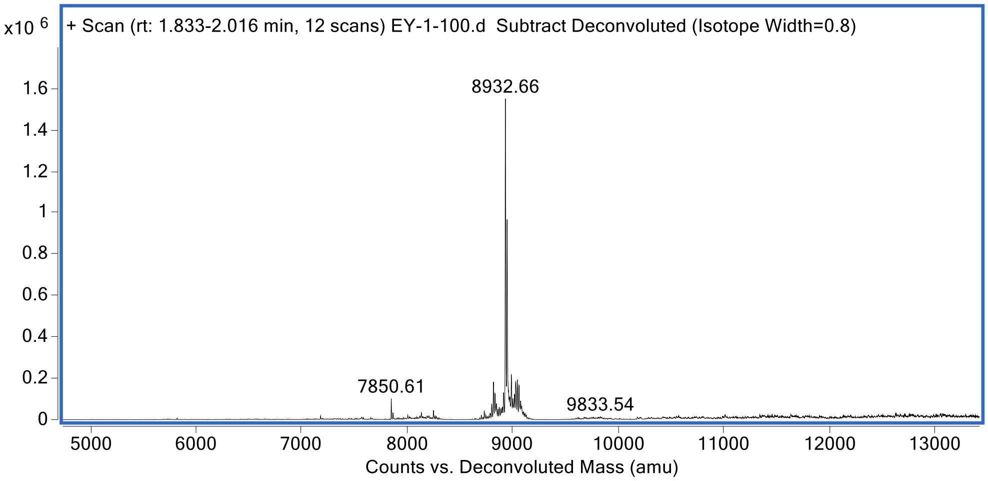

Ac-mISG15$_{CTD}$[R153Agb]-PA

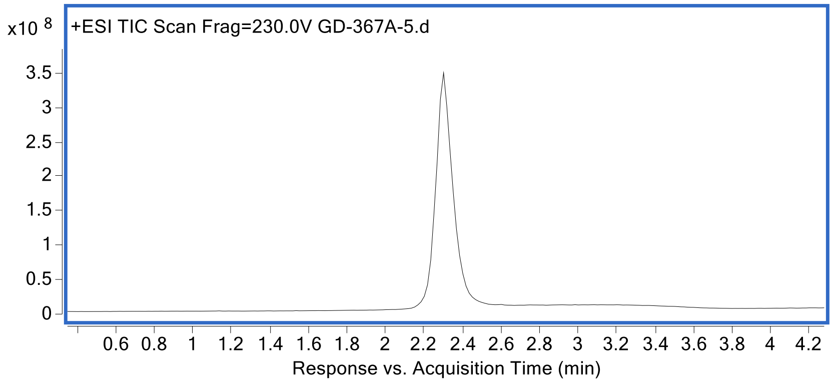

HRMS (ESI): m/z [M] calc. for $C_{399}H_{636}N_{114}O_{116}S$: 8918.2190, found 8934.64.

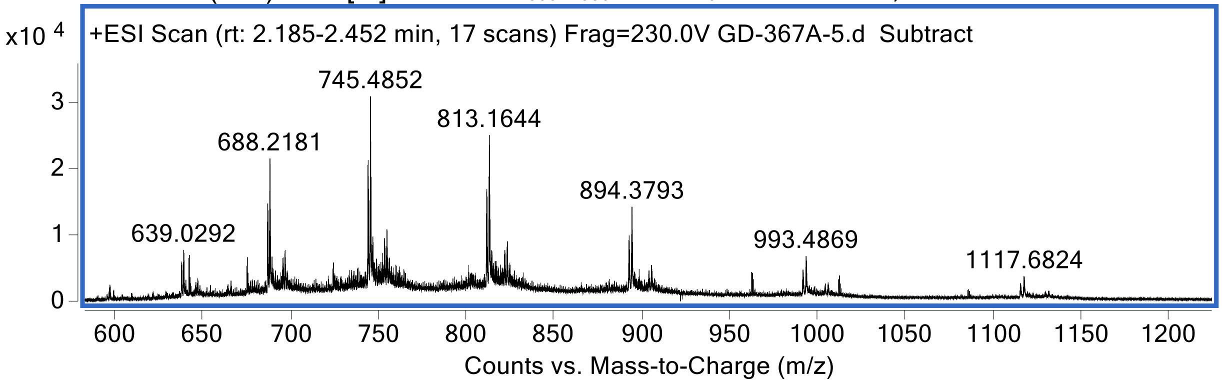

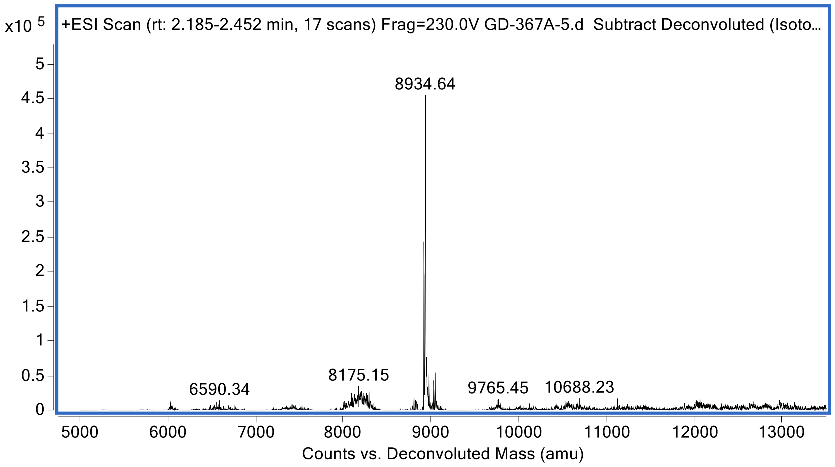

Ac-mISG15$_{CTD}$[R153hR]-PA

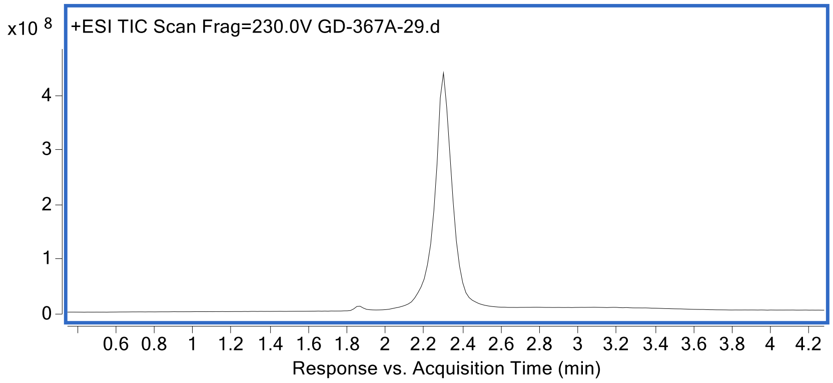

HRMS (ESI): m/z [M] calc. for $C_{401}H_{640}N_{114}O_{116}S$: 8946.2730, found 8962.71.

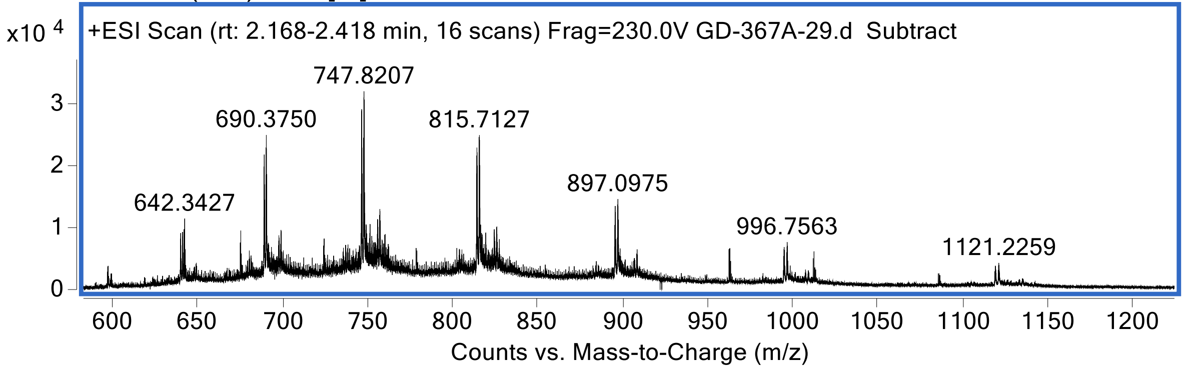

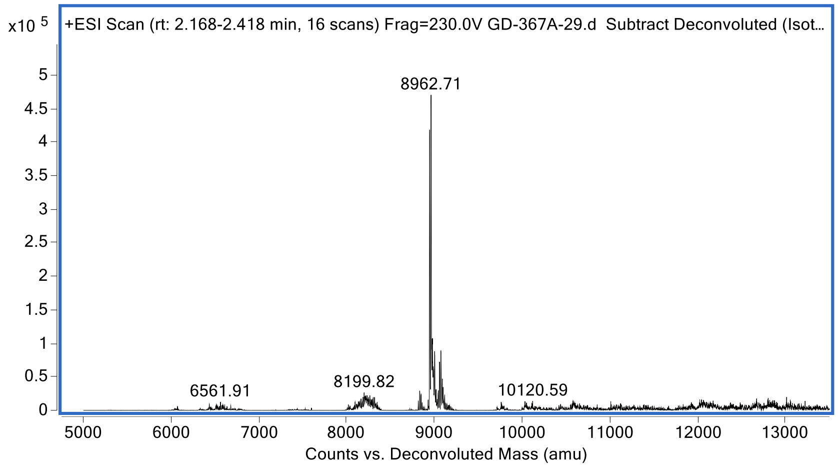

Ac-mISG15$_{CTD}$[R153F(guan)]-PA

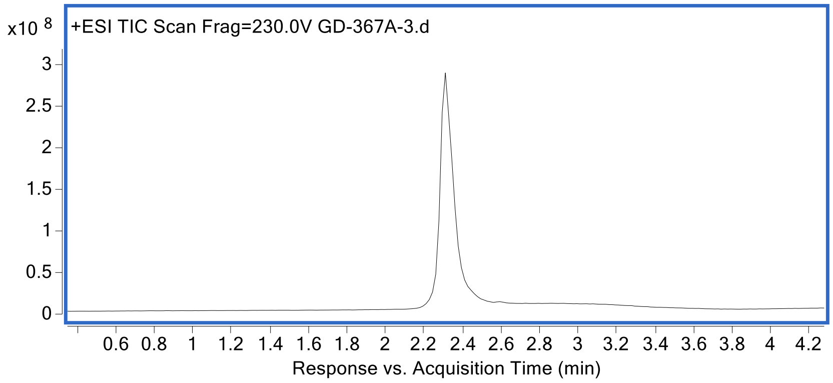

HRMS (ESI): m/z [M] calc. for $C_{404}H_{638}N_{114}O_{116}S$: 8980.2900, found 8980.60.

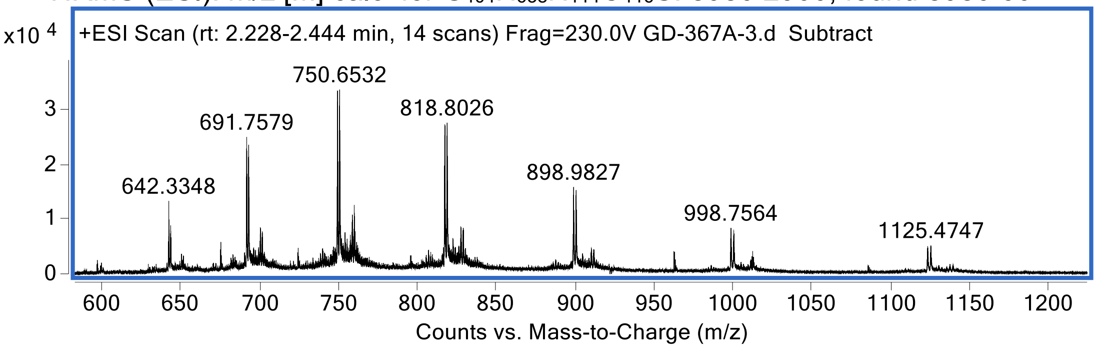

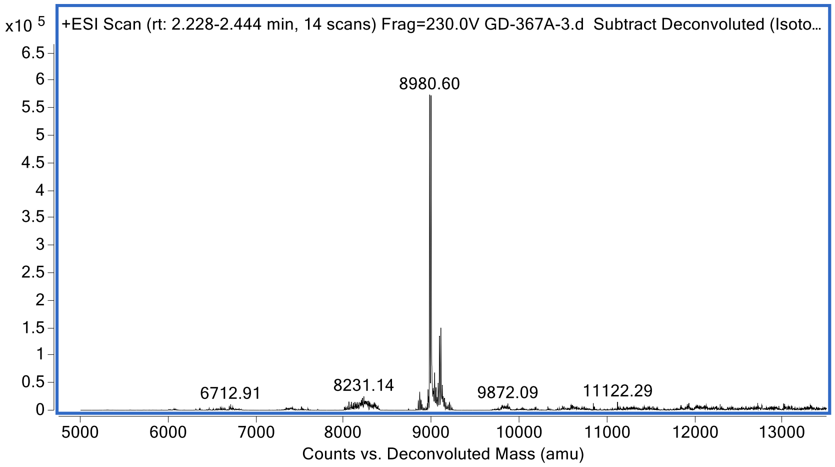

Cy5-mISG15$_{CTD}$[WT]-PA

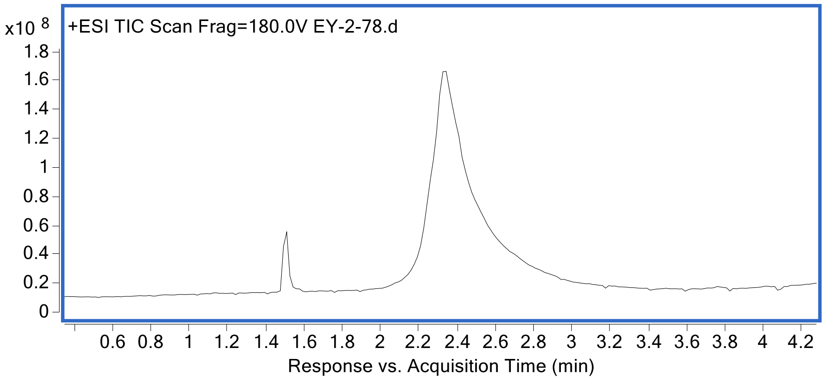

HRMS (ESI): m/z [M] calc. for $C_{430}H_{673}N_{116}O_{116}S$: 9355.8695, found 9370.84.

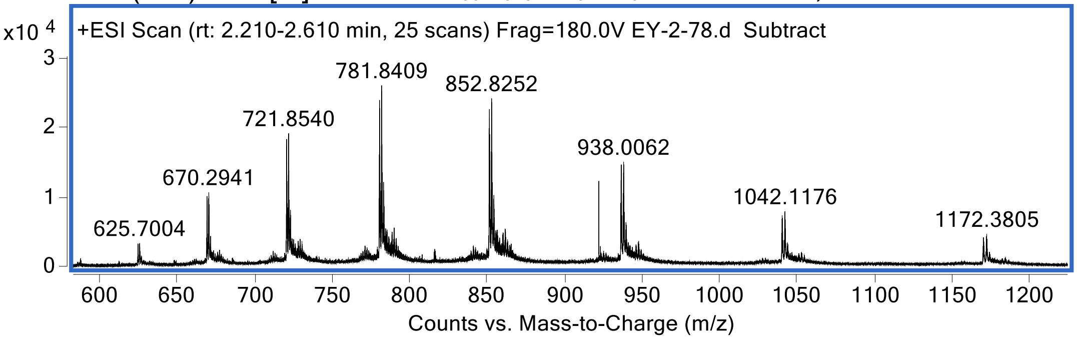

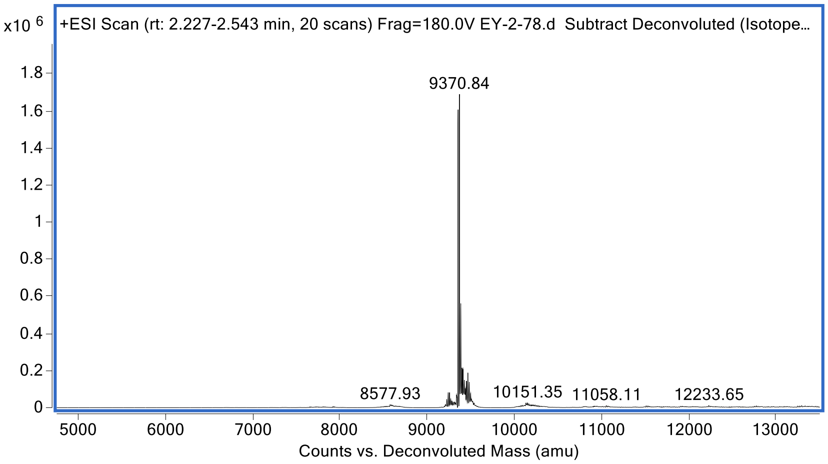

Cy5-mISG15$_{CTD}$[R153Agb]-PA

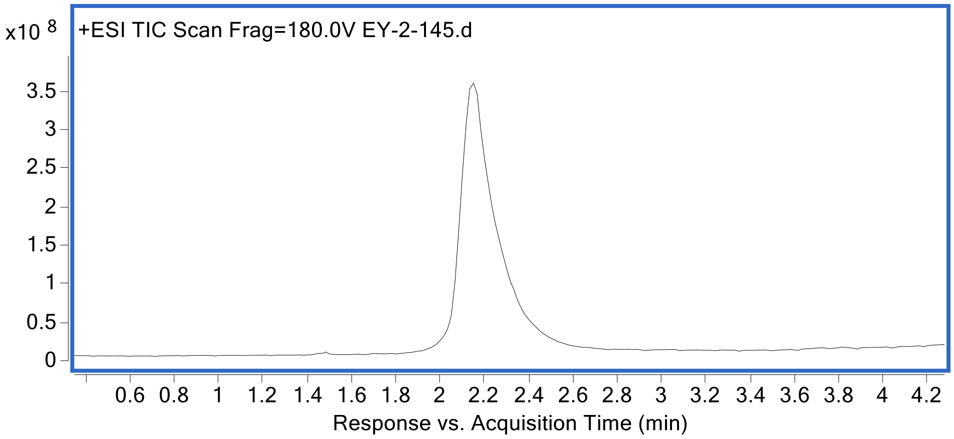

HRMS (ESI): m/z [M] calc. for $C_{429}H_{671}N_{116}O_{116}S$: 9341.8425, found 9341.24.

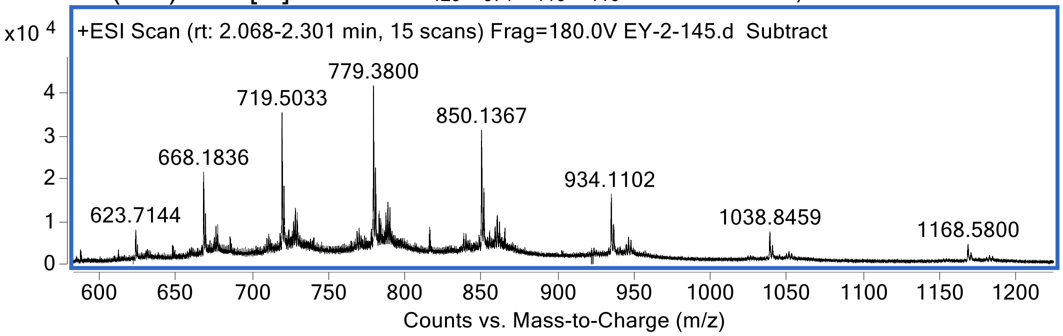

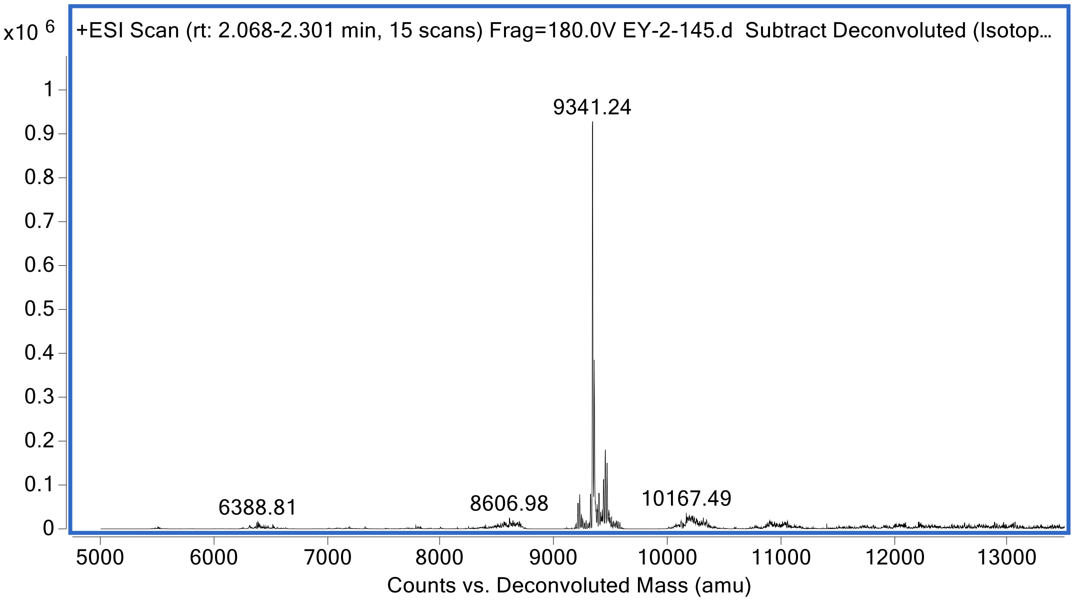

Cy5-mISG15$_{CTD}$[R153F(guan)]-PA

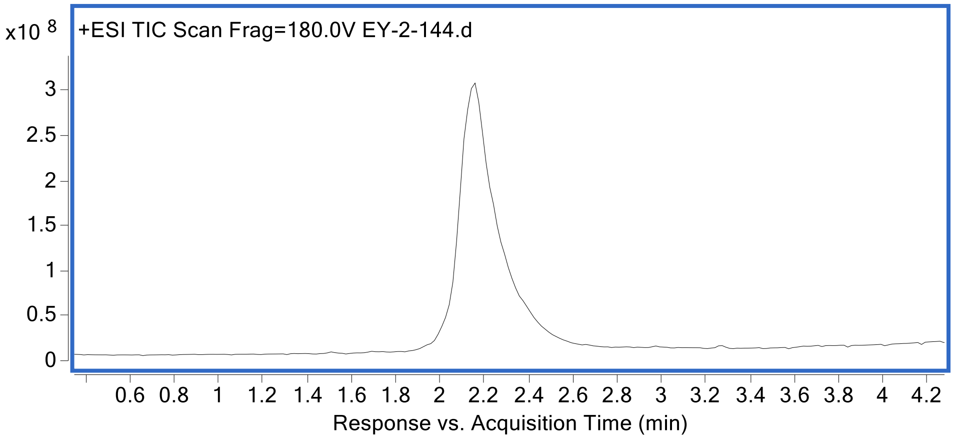

HRMS (ESI): m/z [M] calc. for $C_{434}H_{673}N_{116}O_{116}S$: 9403.9135, found 9403.33.

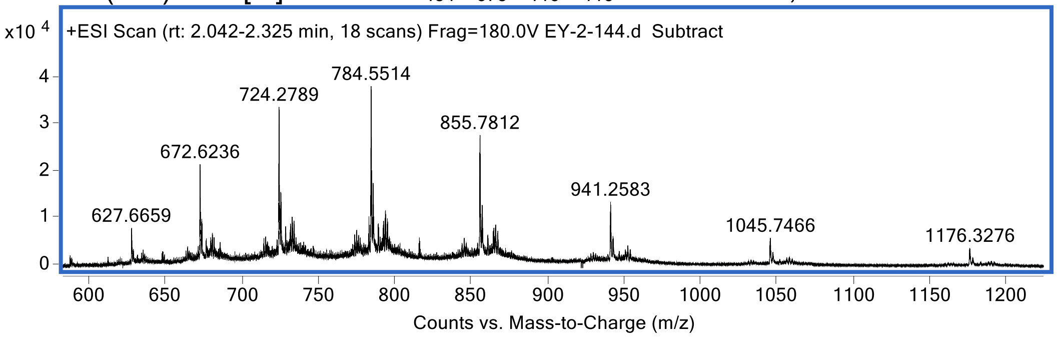

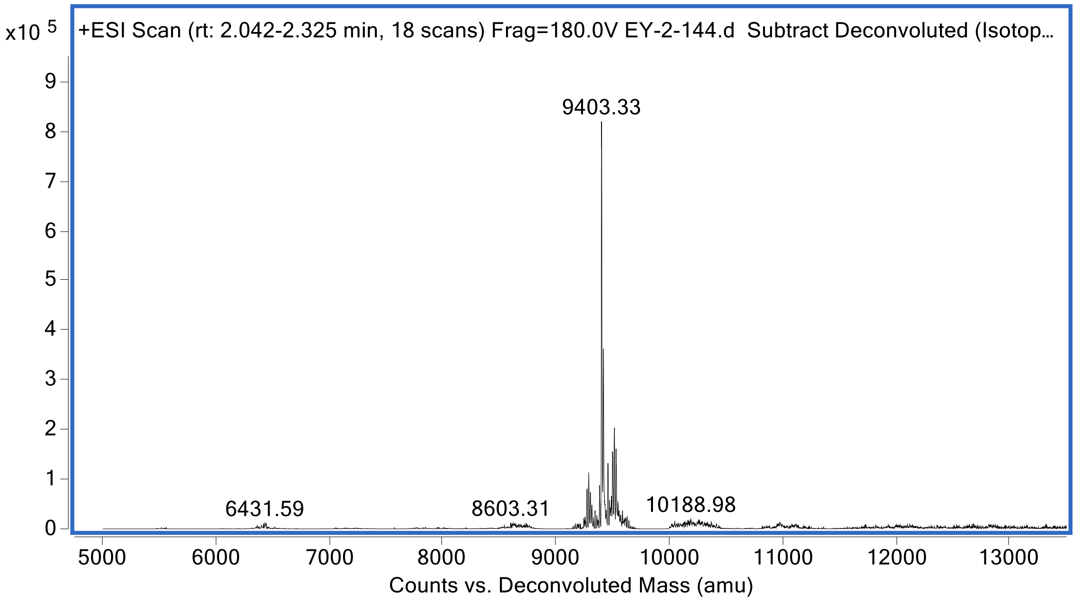

Cy5-mISG15$_{CTD}$[H90F]-PA

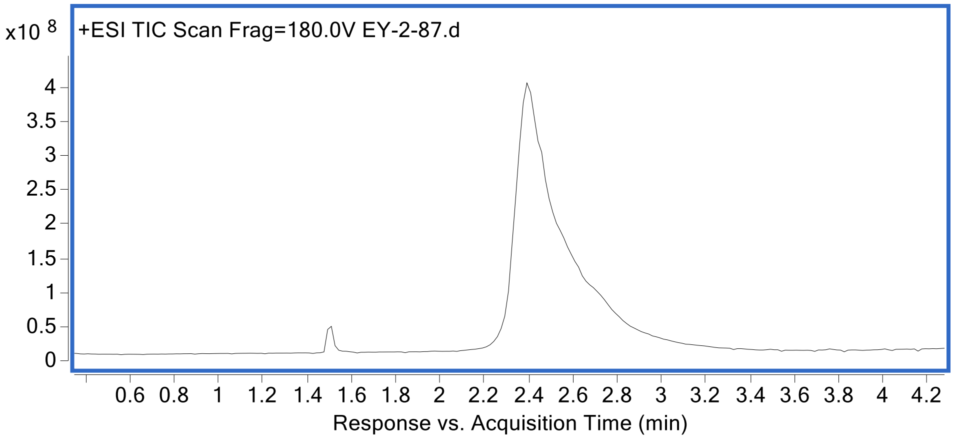

HRMS (ESI): m/z [M] calc. for $C_{433}H_{675}N_{114}O_{116}S$: 9365.9045, found 9381.35.

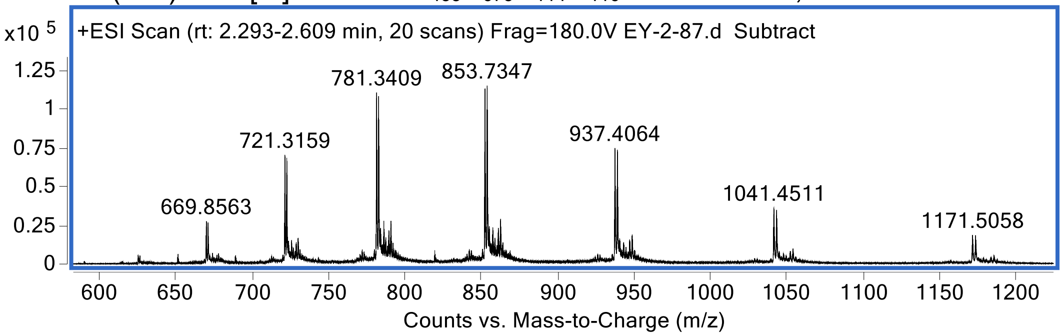

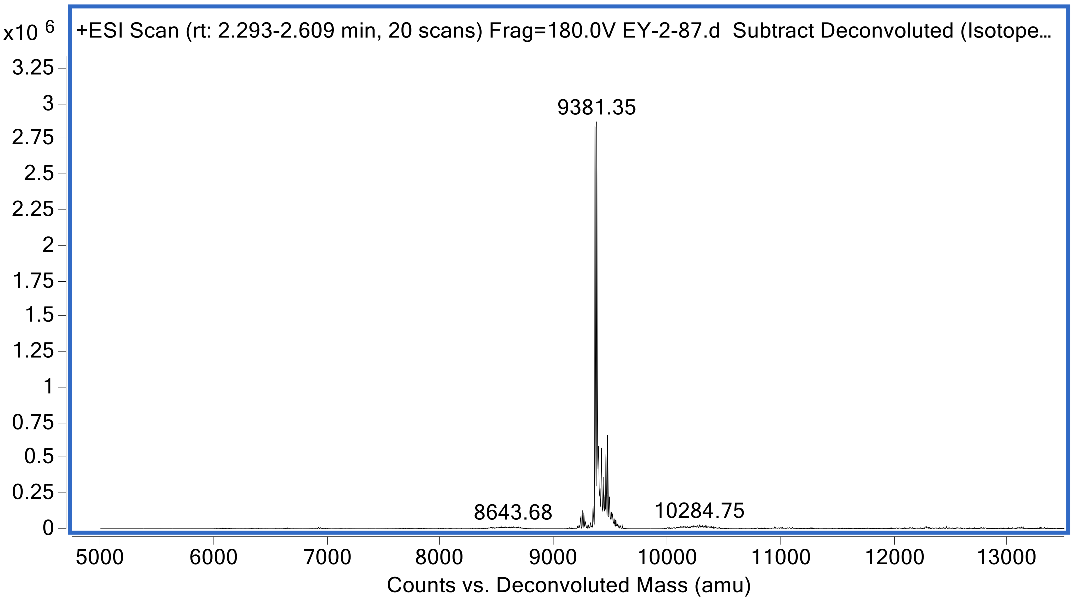

Biotin-mISG15$_{CTD}$[WT]-PA

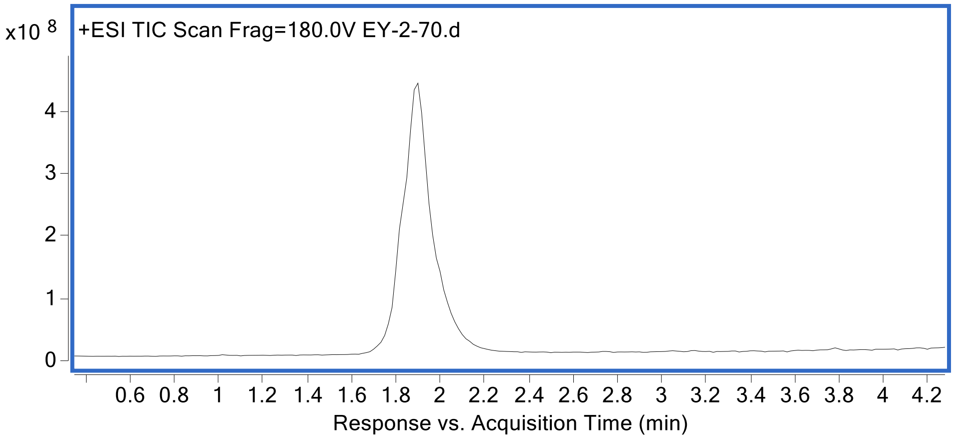

HRMS (ESI): m/z [M] calc. for $C_{414}H_{661}N_{117}O_{118}S_2$: 9229.6630, found 9246.12.

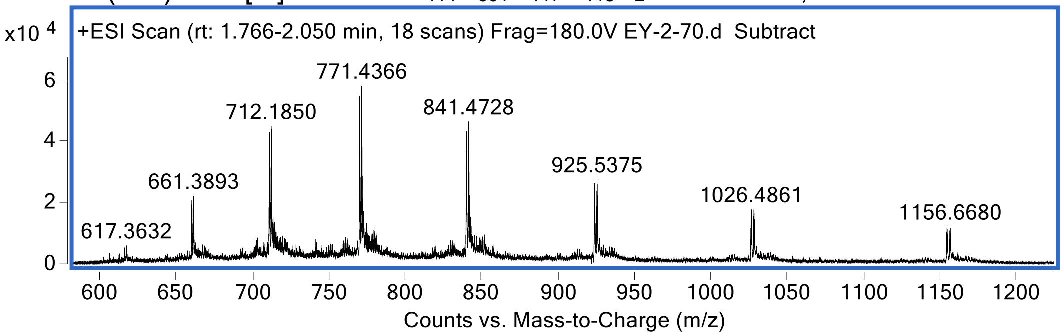

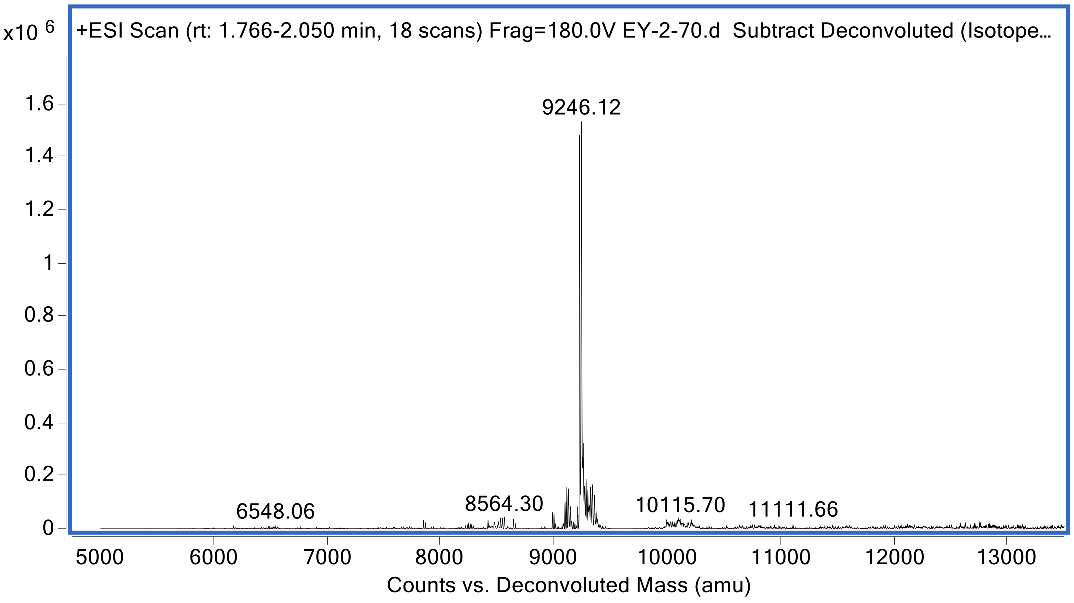

Biotin-mISG15$_{CTD}$[R153Agb]-PA

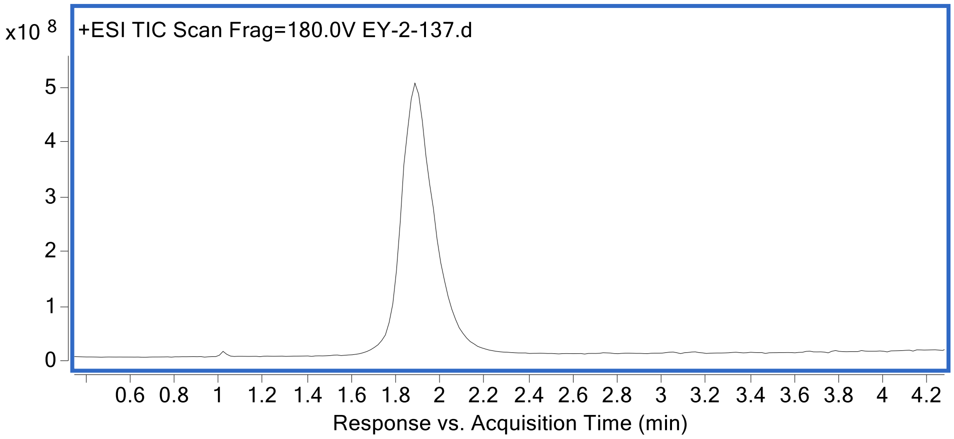

HRMS (ESI): m/z [M] calc. for $C_{413}H_{659}N_{117}O_{118}S_2$: 9215.6360, found 9216.15.

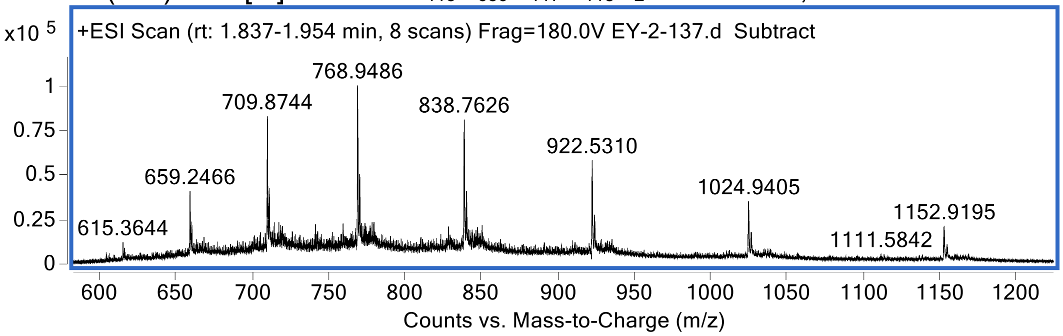

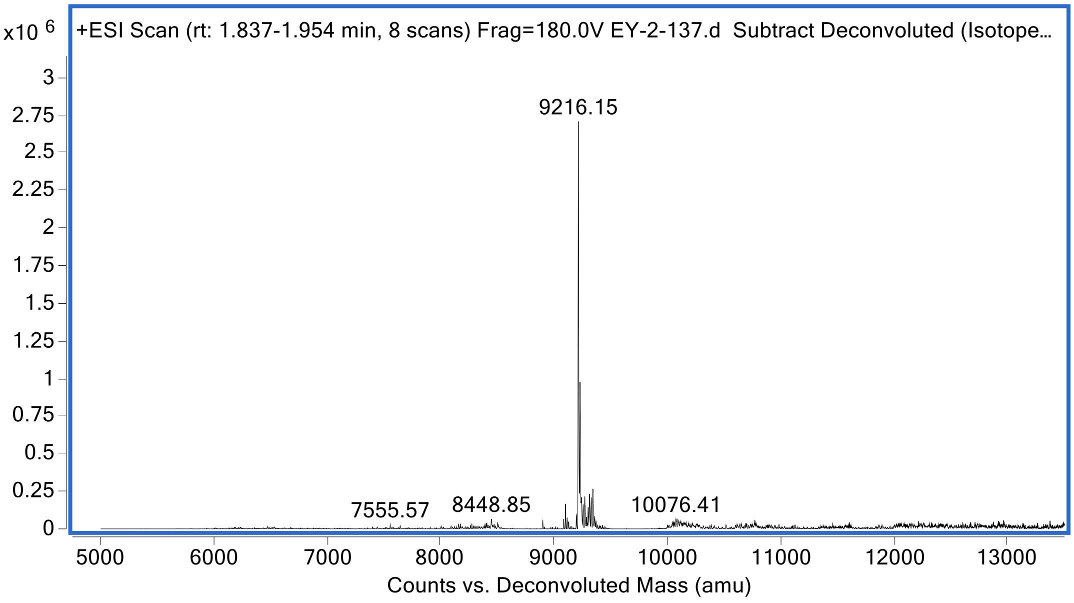

Biotin-mISG15$_{CTD}$[R153F(guan)]-PA

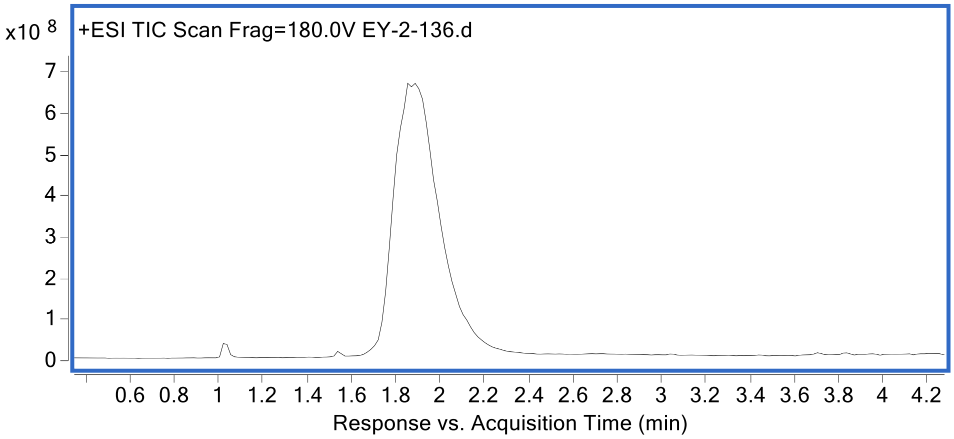

HRMS (ESI): m/z [M] calc. for $C_{418}H_{661}N_{117}O_{118}S_2$: 9277.7070, found 9278.21.

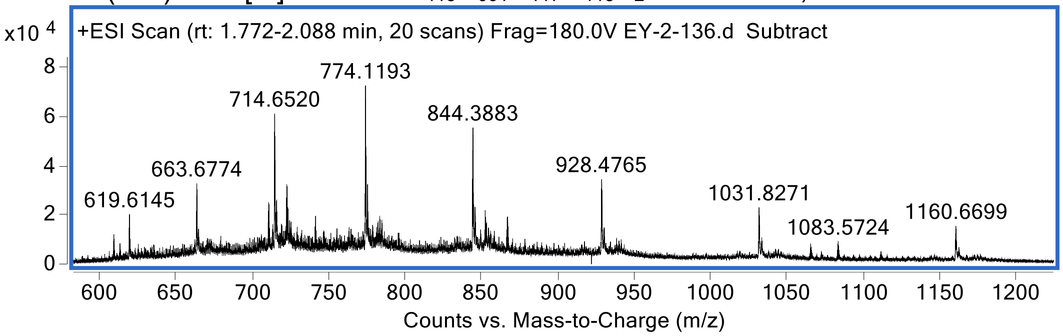

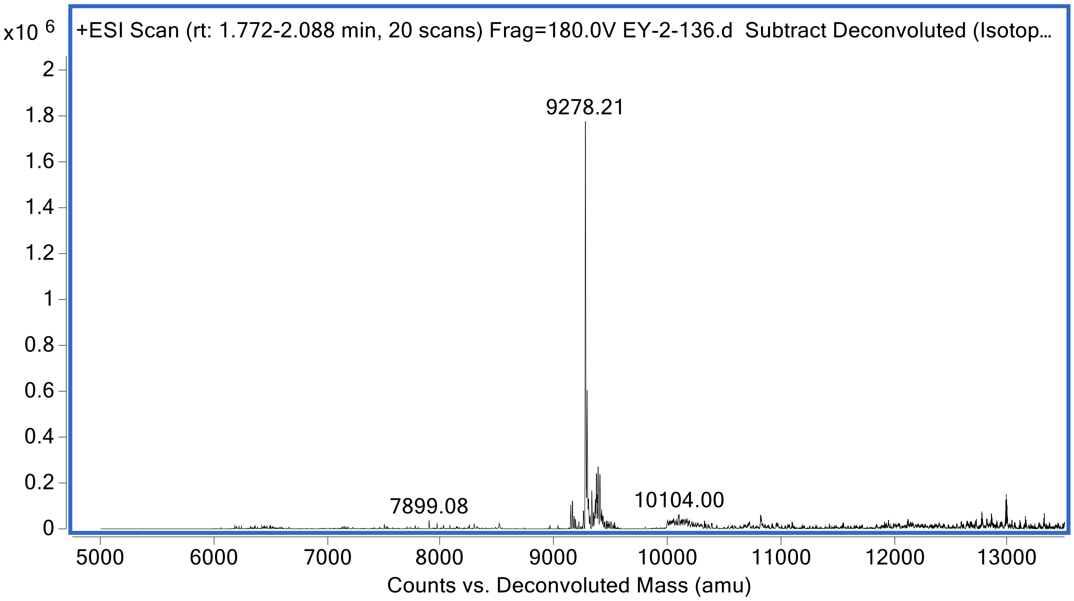

Ac-hISG15$_{CTD}$-VS

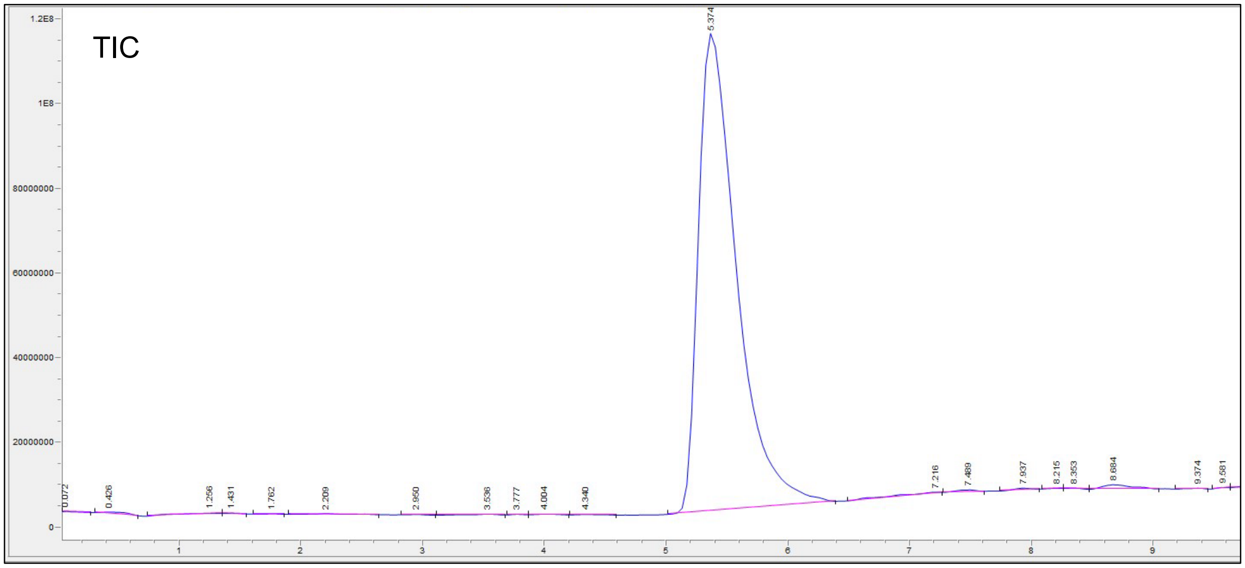

HRMS (ESI): m/z [M] calc. for $C_{388}H_{624}N_{106}O_{114}S_2$: 8662.0080, found 8677.14.

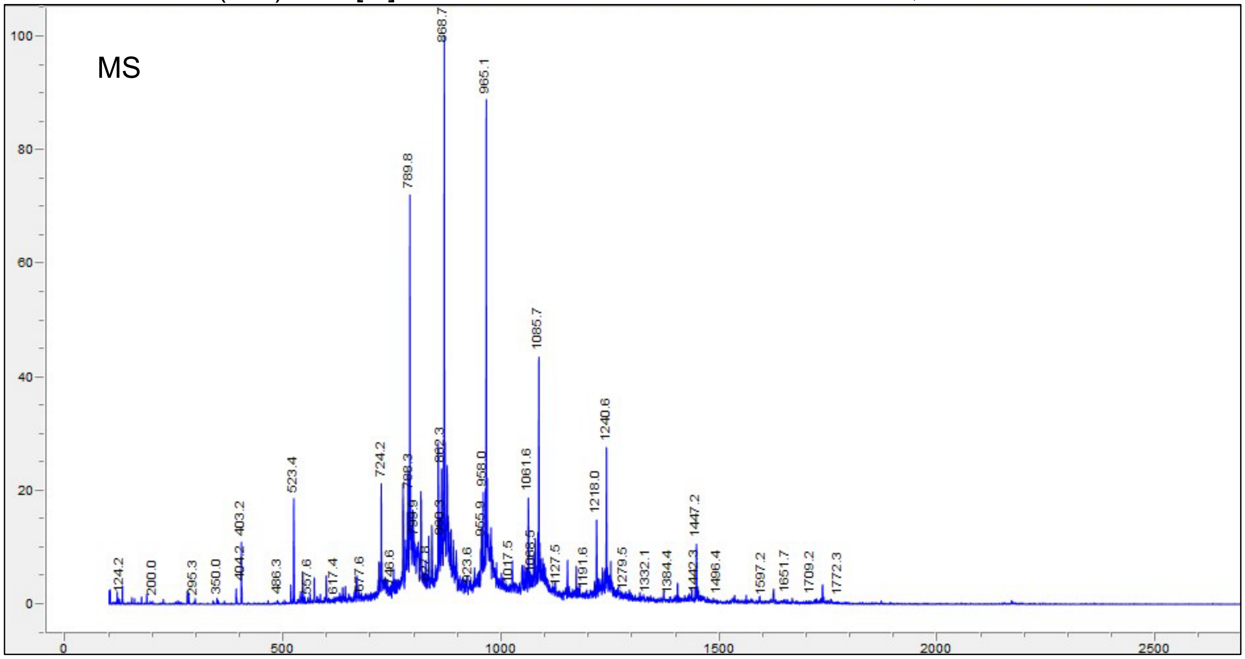

Supplement: Supplementary file 1 — Supplementary information [file 41467_2025_56336_MOESM1_ESM.pdf]
