# Supplementary material for: Chemical tools to define and manipulate interferon-inducible Ubl protease USP18
Source: Nat Commun. 2025 Jan 22;16:957. doi: 10.1038/s41467-025-56336-5 (PMC11754618; doi:10.1038/s41467-025-56336-5)
Supplement: Supplementary file 2 — Reporting Summary [file 41467_2025_56336_MOESM2_ESM.pdf]

nature portfolio

Corresponding author(s): Euna Yoo

Last updated by author(s): 01/07/2025

Reporting Summary

Nature Portfolio wishes to improve the reproducibility of the work that we publish. This form provides structure for consistency and transparency in reporting. For further information on Nature Portfolio policies, see our Editorial Policies and the Editorial Policy Checklist.

Statistics

For all statistical analyses, confirm that the following items are present in the figure legend, table legend, main text, or Methods section.

n/a	Confirmed	
☐	☒	The exact sample size (*n*) for each experimental group/condition, given as a discrete number and unit of measurement
☐	☒	A statement on whether measurements were taken from distinct samples or whether the same sample was measured repeatedly
☐	☒	The statistical test(s) used AND whether they are one- or two-sided<br>*Only common tests should be described solely by name; describe more complex techniques in the Methods section.*
☒	☐	A description of all covariates tested
☒	☐	A description of any assumptions or corrections, such as tests of normality and adjustment for multiple comparisons
☐	☒	A full description of the statistical parameters including central tendency (e.g. means) or other basic estimates (e.g. regression coefficient) AND variation (e.g. standard deviation) or associated estimates of uncertainty (e.g. confidence intervals)
☐	☒	For null hypothesis testing, the test statistic (e.g. *F*, *t*, *r*) with confidence intervals, effect sizes, degrees of freedom and *P* value noted<br>*Give P values as exact values whenever suitable.*
☒	☐	For Bayesian analysis, information on the choice of priors and Markov chain Monte Carlo settings
☒	☐	For hierarchical and complex designs, identification of the appropriate level for tests and full reporting of outcomes
☒	☐	Estimates of effect sizes (e.g. Cohen's *d*, Pearson's *r*), indicating how they were calculated

Our web collection on statistics for biologists contains articles on many of the points above.

Software and code

Policy information about availability of computer code

Data collection	No specialty software was used to collect the data.
Data analysis	Proteome Discoverer 2.4 was used to search proteomics data. BIAevaluation 4.0 was used to analyze the SPR data.

For manuscripts utilizing custom algorithms or software that are central to the research but not yet described in published literature, software must be made available to editors and reviewers. We strongly encourage code deposition in a community repository (e.g. GitHub). See the Nature Portfolio guidelines for submitting code & software for further information.

Data

Policy information about availability of data

All manuscripts must include a data availability statement. This statement should provide the following information, where applicable:

- Accession codes, unique identifiers, or web links for publicly available datasets
- A description of any restrictions on data availability
- For clinical datasets or third party data, please ensure that the statement adheres to our policy

TMT-MS data have been deposited at MassIVE with accession number MSV000094406 (ftp://MSV000094406@massive.ucsd.edu).

Research involving human participants, their data, or biological material

Policy information about studies with human participants or human data. See also policy information about sex, gender (identity/presentation), and sexual orientation and race, ethnicity and racism.

Reporting on sex and gender	No human research participants
Reporting on race, ethnicity, or other socially relevant groupings	No human research participants
Population characteristics	No human research participants
Recruitment	No human research participants
Ethics oversight	No human research participants

Note that full information on the approval of the study protocol must also be provided in the manuscript.

Field-specific reporting

Please select the one below that is the best fit for your research. If you are not sure, read the appropriate sections before making your selection.

☒ Life sciences ☐ Behavioural & social sciences ☐ Ecological, evolutionary & environmental sciences

For a reference copy of the document with all sections, see nature.com/documents/nr-reporting-summary-flat.pdf

Life sciences study design

All studies must disclose on these points even when the disclosure is negative.

Sample size	A sample size for all biological experiments was n=2 or 3.
Data exclusions	Data was not excluded.
Replication	n=2 or 3 independent replicates
Randomization	Randomization was not applicable to this study.
Blinding	No blinding techniques were used in this study, experiments were carried out with labelled compounds.

Reporting for specific materials, systems and methods

We require information from authors about some types of materials, experimental systems and methods used in many studies. Here, indicate whether each material, system or method listed is relevant to your study. If you are not sure if a list item applies to your research, read the appropriate section before selecting a response.

Materials & experimental systems

n/a	Involved in the study	
☐	☒	Antibodies
☐	☒	Eukaryotic cell lines
☒	☐	Palaeontology and archaeology
☒	☐	Animals and other organisms
☒	☐	Clinical data
☒	☐	Dual use research of concern
☒	☐	Plants

Methods

n/a	Involved in the study	
☒	☐	ChIP-seq
☒	☐	Flow cytometry
☒	☐	MRI-based neuroimaging

Antibodies

Antibodies used	USP18 (4813S), USP14 (11931S), GAPDH (5174S), biotin (5597S), ubiquitin (58395S), ISG15 (2743S), FLAG (14793S) antibodies were obtained from Cell Signaling Technology. USP5 (A301-542A) was purchased from Bethyl Laboratories. USP16 antibody (VPA00705) was purchased from Bio-Rad.
Validation	Statement from vendors:<br>4813S: USP18 (D4E7) Rabbit mAb, USP18 (D4E7) Rabbit mAb detects endogenous levels of total USP18 protein. The doublet band

detected by western blot represents full length (39 kDa) and amino-terminal deleted derivative of USP18. Species Reactivity: Human
11931S: USP14 (D8Q6S) Rabbit mAb, USP14 (D8Q6S) Rabbit mAb recognizes endogenous levels of total USP14 protein. Based upon sequence alignment, this antibody is predicted to react with both isoform a and isoform b of USP14. Species Reactivity: Human, Mouse, Rat
A301-542A: Rabbit anti-USP5/IsoT Antibody Affinity Purified, Antibody was affinity purified using an epitope specific to USP5/IsoT immobilized on solid support. The epitope recognized by A301-542A maps to a region between residue 75 and 125 of human ubiquitin specific peptidase 5 (isopeptidase T) using the numbering given in entry CAA62690.1 (GeneID 8078). Species Reactivity: Human, Mouse
VPA00705: RABBIT ANTI USP16, Rabbit anti Human USP16 antibody recognizes the ubiquitin carboxyl-terminal hydrolase 16, also known as deubiquitinating enzyme 16, ubiquitin specific protease 16, ubiquitin thioesterase 16 or ubiquitin-processing protease UBP-M. Rabbit anti Human USP16 antibody detects a band of 112 kDa. The antibody has been extensively validated for western blotting using whole cell lysates. Species Reactivity: Human, Mouse
5174S: GAPDH (D16H11) XP® Rabbit mAb, GAPDH (D16H11) XP® Rabbit mAb detects endogenous levels of total GAPDH protein. Species Reactivity: Human, Mouse, Rat, Monkey
5597S: Anti-biotin (D5A7) Rabbit mAb, Anti-Biotin (D5A7) Rabbit mAb recognizes biotin attached to proteins, peptides, oligonucleotides, or solid matrices. Species Reactivity: Rabbit
58395S: Ubiquitin (P37) Antibody, Ubiquitin (P37) Antibody recognizes endogenous levels of total ubiquitin protein. Species Reactivity: All Species Expected
2743S: ISG15 Antibody, This antibody detects endogenous levels of both free and conjugated ISG15 protein. The antibody does not cross-react with other ubiquitin family members, including ubiquitin, SUMO1, SUMO2, SUMO3 and NEDD8. Species Reactivity: Human, Mouse, Monkey
14793S: DYKDDDDK Tag (D6W5B) Rabbit mAb, DYKDDDDK Tag (D6W5B) Rabbit mAb detects exogenously expressed DYKDDDDK proteins in cells. The antibody recognizes the DYKDDDDK peptide, which is the same epitope recognized by Sigma-Aldrich Anti-FLAG M2 antibody, fused to either the amino-terminus or carboxy-terminus of the target protein. Species Reactivity: All Species Expected

Eukaryotic cell lines

Policy information about cell lines and Sex and Gender in Research

Cell line source(s)

HEK293T (CRL-3216), HeLa (CCL-2), A549 (CCL-185), H1650 (CRL-5883) cells were purchased from American Type Culture Collection (ATCC).

Authentication

Cell lines were not separately authenticated.

Mycoplasma contamination

Not tested.

Commonly misidentified lines
(See ICLAC register)

Commonly misidentified cell lines were not used in this study.

Plants

Seed stocks

Report on the source of all seed stocks or other plant material used. If applicable, state the seed stock centre and catalogue number. If plant specimens were collected from the field, describe the collection location, date and sampling procedures.

Novel plant genotypes

Describe the methods by which all novel plant genotypes were produced. This includes those generated by transgenic approaches, gene editing, chemical/radiation-based mutagenesis and hybridization. For transgenic lines, describe the transformation method, the number of independent lines analyzed and the generation upon which experiments were performed. For gene-edited lines, describe the editor used, the endogenous sequence targeted for editing, the targeting guide RNA sequence (if applicable) and how the editor was applied.

Authentication

Describe any authentication procedures for each seed stock used or novel genotype generated. Describe any experiments used to assess the effect of a mutation and, where applicable, how potential secondary effects (e.g. second site T-DNA insertions, mosiacism, off-target gene editing) were examined.
